# Apoptotic vesicles restore liver macrophage homeostasis to counteract type 2 diabetes

**DOI:** 10.1002/jev2.12109

**Published:** 2021-05-24

**Authors:** Chenxi Zheng, Bingdong Sui, Xiao Zhang, Jiachen Hu, Ji Chen, Jin Liu, Di Wu, Qingyuan Ye, Lei Xiang, Xinyu Qiu, Siying Liu, Zhihong Deng, Jun Zhou, Shiyu Liu, Songtao Shi, Yan Jin

**Affiliations:** ^1^ State Key Laboratory of Military Stomatology & National Clinical Research Center for Oral Diseases & Shaanxi International Joint Research Center for Oral Diseases Center for Tissue Engineering School of Stomatology The Fourth Military Medical University Xi'an Shaanxi China; ^2^ South China Center of Craniofacial Stem Cell Research Guanghua School and Hospital of Stomatology Sun Yat‐sen University Guangzhou Guangdong China; ^3^ Department of Prosthodontics National Laboratory for Digital and Material Technology of Stomatology Beijing Key Laboratory of Digital Stomatology National Clinical Research Center for Oral Diseases Peking University School and Hospital of Stomatology Beijing China; ^4^ Department of Anatomy and Cell Biology School of Dental Medicine University of Pennsylvania Philadelphia Pennsylvania USA

**Keywords:** apoptotic vesicles, calreticulin, efferocytosis, macrophages, mesenchymal stem cells, type 2 diabetes

## Abstract

Apoptosis is a naturally occurring process generating plenty of apoptotic vesicles (apoVs), but the feature, fate and function of apoVs remain largely unknown. Notably, as an appealing source for cell therapy, mesenchymal stem cells (MSCs) undergo necessary apoptosis and release apoVs during therapeutic application. In this study, we characterized and used MSC‐derived apoVs to treat type 2 diabetes (T2D) mice, and we found that apoVs were efferocytosed by macrophages and functionally modulated liver macrophage homeostasis to counteract T2D. We showed that apoVs can induce macrophage reprogramming at the transcription level in an efferocytosis‐dependent manner, leading to inhibition of macrophage accumulation and transformation of macrophages towards an anti‐inflammation phenotype in T2D liver. At the molecular level, we discovered that calreticulin (CRT) was exposed on the surface of apoVs to act as a critical ‘eat‐me’ signal mediating apoV efferocytosis and macrophage regulatory effects. Importantly, we demonstrated that CRT‐mediated efferocytosis of MSC‐derived apoVs contributes to T2D therapy with alleviation of T2D phenotypes including glucose intolerance and insulin resistance. These findings uncover that functional efferocytosis of apoVs restores liver macrophage homeostasis and ameliorates T2D.

## 1 INTRODUCTION

Billions of cells undergo apoptosis per day in the human body as an essential metabolic activity to maintain physiological homeostasis, during which a large number of apoptotic vesicles (apoVs) are produced (Caruso & Poon, 2018; Grant et al., 2019; Kakarla et al., 2020). While previous studies on apoVs mainly focused on apoptotic bodies (apoBDs) (1‐5 μm in diameter), an increasing number of research have identified smaller membrane‐bound vesicles secreted during apoptosis including apoptotic microvesicles (apoMVs) (0.1‐1 μm in diameter) and apoptotic exosomes (apoExos) (< 150 nm in diameter), together forming the apoV populations (Poon et al., 2019). The production of apoVs is a highly controlled process resulting in load of various functional cargos, but a very limited number of these molecules has been functionally characterized (Brock et al., 2019; Pavlyukov et al., 2018; Zernecke et al., 2009). ApoVs are emerging as critical players in multiple physiological and pathophysiological settings (Brock et al., 2019; Dieudé et al., 2015; Liu et al., 2018; Pavlyukov et al., 2018; Zernecke et al., 2009), whereas most studies have focused on the function of extracellular vesicles (EVs) derived from viable cells. The feature, fate and function of apoVs are not fully understood.

It is generally recognized that macrophages, especially those within the liver, are the main ‘professional’ phagocytes responsible for clearance of apoptotic cells, constituting an evolutionarily conserved process known as efferocytosis (Boada‐Romero et al., 2020; Doran et al., 2020; Morioka et al., 2019). This orchestrated process not only removes apoptotic cells from tissues but also generates imperative immunosuppressive effects *via* actively eliciting anti‐inflammation responses in macrophages (Perry et al., 2019; Zhang et al., 2019). Despite the identification of apoVs as smaller and more easily engulfed cell fragments (Grant et al., 2019), there are few studies investigating the functional efferocytosis of apoVs by macrophages. Macrophages are a vital cellular component of the liver with critical roles in homeostasis maintenance as well as disease occurrence and progression (Krenkel & Tacke, 2017; Tacke, 2017). In specific, excessive infiltration and pro‐inflammatory activation of macrophages lead to chronic low‐grade inflammation that contributes to a spectrum of diseases, including type 2 diabetes (T2D) (Castegna et al., 2020; Mcnelis & Olefsky, 2014), a chronic progressive disease with high prevalence around the world (Defronzo et al., 2015; Saeedi et al., 2019; Zheng et al., 2018). It is important to reveal the effects of apoV efferocytosis on macrophages to establish a potential immunomodulatory treatment for T2D.

As an appealing cell source for therapies, mesenchymal stem cells (MSCs) possess potent immunoregulatory and anti‐inflammation abilities, which have shown therapeutic potential in various immunological and inflammatory diseases, including T2D (Galipeau & Sensébé, 2018; Shi et al., 2018; Wang et al., 2014; Zang et al., 2017). Intriguingly, MSCs have been uncovered to undergo apoptosis after in vivo application, which contributes to the therapeutic efficacy of MSCs (Galleu et al., 2017; Liu et al., 2014; Weiss et al., 2019). We have demonstrated that local application of MSC‐derived apoVs alleviated myocardial infarction *via* regulating recipient endothelial cells (Liu et al., 2020) and that systemic infusion of MSC‐derived apoVs ameliorated osteopenia *via* rescuing endogenous MSCs (Liu et al., 2018). Nevertheless, the in vivo fate, downstream biological responses, and implications of MSC‐derived apoVs efferoctytosed by macrophages are largely unknown.

In this study, we characterized the proteomic profiles of MSC‐derived apoVs *via* LC‐MS/MS analysis and identified enrichment of various functional proteins, which extended our understanding of apoVs. We showed that efferocytosis of apoVs induced transcriptional reprogramming of macrophages in vitro and inhibited diseased liver macrophage infiltration and activation in vivo. Mechanistically, we demonstrated that calreticulin (CRT) was exposed on the surface of apoVs, which acted as a pivotal ‘eat‐me’ signal mediating the efferocytosis of apoVs by macrophages and the regulatory effects. Moreover, we uncovered that engulfment of apoVs *via* CRT improved glucose tolerance, alleviated insulin resistance and ameliorated hepatic steatosis in high fat diet (HFD)‐induced T2D. Taken together, our findings reveal that efferocytosis of MSC‐derived apoVs re‐establishes liver macrophage homeostasis, thus possessing potent therapeutic potential in metabolic diseases.

## 2 MATERIALS AND METHODS

### 2.1 Animals

Animal experiments were performed in accordance with the Guidelines of Institutional Animal Care and Use Committee of the Fourth Military Medical University and the ARRIVE guidelines. C57BL/6 mice were purchased from the Laboratory Animal Center of the Fourth Military Medical University. *Fas* mutant (*Fas^mut^*) mice (B6.MRL‐*Fas^lpr^*/J, JAX# 000482) and wild‐type (WT) mice (JAX# 000664) were purchased from the Jackson Laboratory (Liu et al., 2018). Mice were maintained under specific pathogen‐free conditions (24℃, 12 h light/dark cycles and 50% humidity), and were kept feeding and drinking ad libitum.

For C57BL/6 mice, six‐week‐old male mice were placed on a HFD (60% kcal from fat; D12492, Research Diets, USA) or a normal chow diet until the end of the experimental protocol. After 8 weeks of HFD feeding, mice were intravenously administrated with apoVs (appropriately 200 μg on the basis of protein measurement) once a week for 4 weeks. Metabolic assays were conducted at the indicated times. At week 20, mice were sacrificed, and the liver and blood were sampled.

For *Fas^mut^* and WT mice, six‐week‐old male mice were placed on HFD until the end of the experimental protocol and administrated with apoVs biweekly. Metabolic assays were conducted at the indicated times. At week 16, mice were sacrificed, and the liver and blood were sampled.

### 2.2 Isolation, culture and characterization of MSCs

The use of human bone marrow samples was approved by the Ethics Committee of the Fourth Military Medical University with informed consent of the donors. Briefly, MSCs were isolated from the bone marrow aspirates of the iliac crest and purified using the Percoll density gradient centrifugation method, as formerly reported (Liu et al., 2018). Primary MSCs were cultured in alpha‐Minimum Essential Medium (α‐MEM) (Gibco, USA) supplemented with 10% fetal bovine serum (FBS) (Gibco, USA), 2 mM L‐glutamine (Invitrogen, USA) and 1% penicillin/streptomycin (Invitrogen, USA) at 37℃ in a humidified atmosphere of 5% CO_2_. When they were 80%–90% confluent, the adherent cells were digested with 0.25% trypsin (MP Biomedicals, USA) and passaged in vitro. MSCs at the third and fourth passages were used for all the experiments.

MSCs were identified in accordance with previous studies (Liao et al., 2017; Liu et al., 2018). For flow cytometric analysis of the surface markers, MSCs were harvested and suspended in phosphate buffered saline (PBS) supplemented with 2% FBS at 5×10^6^ cells/ml. MSCs were incubated with PE‐conjugated anti‐CD90 (12‐0909‐41, eBioscience, USA; diluted at 1:20), PE‐conjugated anti‐CD105 (800504, BioLegend, USA; diluted at 1:20), FITC‐conjugated anti‐CD73 (344015, BioLegend, USA; diluted at 1:20), PE‐conjugated anti‐CD45 (304008, BioLegend, USA; diluted at 1:20), PE‐conjugated anti‐CD34 (343506, BioLegend, USA; diluted at 1:20) and PerCP/Cy5.5‐conjugated anti‐CD11b (101227, BioLegend, USA; diluted at 1:100) at 4℃ for 30 min. The percentages of positively stained cells were analyzed by a CytoFLEX flow cytometer (Beckman Coulter, USA) and FlowJo 10.0 software (Flow Jo LLC, USA). For colony formation, MSCs were harvested and seeded at a density of 2×10^3^ cells/dish in 5 cm culture dishes. After 14 days, MSCs were fixed with 4% paraformaldehyde (PFA) (Sigma‐Aldrich, USA) and stained with 0.1% crystal violet (Sigma‐Aldrich, USA) for colonies with over 50 cells. For cell morphological observation, MSCs were cultured at a density of 2×10^5^ cells/well in 6‐well plates for 3 days, and the cell morphology was estimated. For osteogenic differentiation, the seeded MSCs were further cultured in osteogenic medium containing 10 mM β‐glycerophosphate, 50 μg/ml ascorbic acid and 10 nM dexamethasone (all from Sigma‐Aldrich, USA). After 21 days, alizarin red S (Sigma‐Aldrich, USA) staining was performed to determine mineralization. For adipogenic differentiation, the seeded MSCs were further cultured in adipogenic medium containing 0.5 mM isobutylmethylxanthine, 1 μM dexamethasone, 10 μM insulin and 0.2 mM indomethacin (all from Sigma‐Aldrich, USA). After 14 days, oil red O (ORO) (Sigma‐Aldrich, USA) staining was performed to determine the lipid droplet formation. The MSCs used for apoV isolation also underwent alizarin red S and ORO staining to confirm the non‐differentiated phenotype before use. Photographs were taken using an inverted optical microscope (Olympus, Japan).

### 2.3 Induction of MSC apoptosis

Undifferentiated MSCs were washed twice with PBS and the culture medium was substituted by a complete medium containing EV‐depleted FBS and 250 nM staurosporine (STS) (Cell Signaling Technology, USA). EV‐depleted FBS was obtained by ultracentrifugation at 100,000 g for 18 h which prevented contamination of apoVs by FBS‐derived EVs. After 12 h treatment, the apoptosis of MSCs was detected by morphological observation and terminal deoxynucleotidyl transferase dUTP nick end labeling (TUNEL) staining. TUNEL staining was performed with the One Step TUNEL Apoptosis Assay Kit (Beyotime Biotechnology, China) according to the manufacturer's instruction, with counterstaining by Hoechst 33342 (Sigma‐Aldrich, USA).

### 2.4 Collection and identification of apoVs

ApoVs were collected according to an optimized protocol (Liu et al., 2020). Briefly, the supernatant of apoptotic MSCs was collected at the 12th hour mark of MSCs induced for apoptosis and subsequently centrifuged at 800 g for 10 min. The supernatant was further collected and centrifuged at 16,000 g for 30 min to obtain apoVs which were then washed twice with filtered PBS. ApoVs were quantified by measuring the protein concentration *via* a BCA Protein Assay Kit (TIANGEN, China).

For transmission electron microscope (TEM) observation, apoV pellet was resuspended in 2% PFA, and 20 μl apoVs were deposited on 200‐mesh formvar‐coated copper grids and dried at room temperature for 5 min. After removing excess suspension using filter paper, the apoVs were negatively stained with uranyl acetate (Sigma‐Aldrich, USA) at room temperature for 2 min, washed with distilled water and dried. Imaging was performed under a FEI Tecnai G2 Spirit Biotwin TEM (Thermo Fisher, USA) operating at 100 kV, with a PHURONA camera (EMSIS, Germany) and RADIUS 2.0 software (EMSIS, Germany).

For cryo‐electron microscopy (Cryo‐EM) observation, approximately 3 μl apoVs at 1 μg/μl were applied to freshly glow‐discharged Quatifoil (300 mesh R1.2/1.3) holey carbon grids. After removing excess solution with filter paper, the grids were plunged rapidly into liquid ethane bath cooled with liquid nitrogen using a semi‐automatic Vitrobot Mark IV (Thermo Fisher, USA) with blotting force of level ‐1 and blotting time of 0.5 s at 4°C, 100% humidity. The images were collected on a Talos Arctica cryo‐electron microscope (Thermo Fisher, USA) with a Ceta camera. Each micrograph was exposed for 1 s at a dose rate of 40 e/pixel/s. The pixel size at the object scale is 1.584 Å (nominal magnification 92 K) and 2.557 Å, and the defocus is about ‐3 μm.

For size distribution evaluation, nanoparticle tracking analysis (NTA) was performed by a NanoSight NS300 (Malvern, UK) equipped with a 405 nm laser and a sCMOS camera. Resuspended apoVs were diluted 50‐fold in filtered PBS to achieve a final concentration of 3.2×10^10^ particle/ml. The capture length was 60 s with camera level set to 14 and detection threshold set to 3. The image of filtered PBS was taken to verify that the diluent had no particle in it. A total of 1498 frames were captured and analyzed. The NTA 3.2 Dev Build 3.2.16 software (Malvern, UK) was used for capturing and data analysis.

For apoptotic marker detection, purified apoVs were characterized by western blotting using anti‐Caspase‐3 and anti‐β‐Actin antibodies, as stated below. Additionally, apoVs were stained with Annexin‐V‐Fluos labeling reagent (Roche, Germany) followed by observation under a confocal microscope (FV1000, Olympus, Japan) and detection *via* a ACEA NovoCyte flow cytometer (ACEA Biosciences, USA).

### 2.5 Proteomic analysis

Protein lysates of MSCs and apoVs were prepared and subjected to LC‐MS/MS analysis on a TripleTOF 5600 mass spectrometer (ABSCIEX, USA) with a NanoSpray III ion source (ABSCIEX, USA). The raw data were analyzed using MaxQuant 1.5.3.30 software with the Andromeda search engine (Cox & Mann, 2008). Proteins were identified by comparing against the Uniprot database with false discovery rate (FDR) set at 0.01 for both peptides and proteins. Proteins were quantified using the default parameters in MaxQuant. Among the identified proteins, 481 proteins were differentially expressed (DEPs) (Fold change > 1.5 and *Q* value < 0.05). Proteins that were significantly upregulated in apoVs were included for further functional analysis based on Gene Ontology (GO) and Kyoto Encyclopedia of Genes and Genomes (KEGG) databases. The details of all the identified proteins were listed in Supplementary Table 1.

### 2.6 Culture and characterization of bone marrow‐derived macrophages (BMDMs)

For culture of BMDMs, bone marrow cavities were flushed with PBS which was then passed through a cell strainer and subjected to red blood cell lysis as previously described (Dou et al., 2020). Freshly isolated cells were cultured in high‐glucose DMEM (Gibco, USA) supplemented with 10% FBS (Gibco, USA), 2 mM L‐glutamine (Invitrogen, USA), 1% penicillin/streptomycin (Invitrogen, USA) and 20 ng/ml recombinant mouse macrophage colony‐stimulating factor (M‐CSF) (PeproTech, USA). After induction for 7 days, mature BMDMs were collected and identified by flow cytometry with Alexa Fluor 488‐conjugated anti‐F4/80 (123120, BioLegend, USA; diluted at 1:400) and PerCP/Cy5.5‐conjugated anti‐CD11b antibodies (101227, BioLegend, USA; diluted at 1:100). Mature BMDMs were used for subsequent assays.

### 2.7 ApoV uptake by BMDMs in vitro

To detect the uptake of apoVs by BMDMs in vitro, apoVs were labelled with PKH26 or PKH67 (both from Sigma‐Aldrich, USA) following the manufacturer's instructions. In specific, after PKH staining, apoV suspension was added by an equal volume of EV‐depleted FBS and incubated for 1 min to allow binding of excess dye. Then, apoVs were isolated *via* centrifugation and washed twice with PBS to further get rid of unbound PKH. In order to prove that unbound PKH has been removed, the stained apoVs were resuspended in PBS and underwent centrifugation, after which the supernatant was used as the negative control (NC), and the apoV pellets were resuspended and added to cultured BMDMs at indicated time points and concentrations (Dou et al., 2020).

After washing with PBS for 3 times, BMDMs incubated with NC suspension or PKH26‐labeled apoVs were fixed with 4% PFA, blocked at room temperature for 30 min, and incubated with anti‐F4/80 antibody (ab6640, Abcam, UK; diluted at 1:400) at 4℃ overnight. After washing with PBS for 3 times, the cells were incubated with FITC‐conjugated anti‐rat secondary antibody (Molecular Probes, USA) at room temperature for 1.5 h. Then, the cells were washed for 3 times with PBS and the nuclei were counterstained with Hoechst 33342 (Sigma‐Aldrich, USA). Fluorescence imaging was performed by a confocal microscope (FV1000, Olympus, Japan) and analyzed using the ImageJ software. Besides, flow cytometric analysis was performed on BMDMs treated with NC suspension or PKH67‐labeled apoVs.

### 2.8 RNA sequencing (RNA‐seq) analysis

BMDMs were treated with apoVs at 20 ng/ml for 24 h and then washed with PBS for 3 times. Total RNA was isolated from control BMDMs and apoV‐treated BMDMs using Trizol (Invitrogen, USA) according to the manual instruction. RNA sequencing libraries were generated with an insert size ranging from 100 to 500 bp, and sequenced using the BGISEQ‐500 platform (BGI‐Shenzhen, China). Clean reads were obtained by filter with SOAPnuke 1.5.2 software (BGI‐Shenzhen, China) according to the parameter ‐l 15 ‐q 0.2 ‐n 0.05 (Chen et al., 2018). Each sample produced 6.68 G data on average. The clean reads were mapped to the reference genome using HISAT2 2.0.4 software. Data processing and analysis was performed using the R programming language. In specific, mapped reads were used for calculation of gene expression levels by RSEM 1.2.12 software (Li & Dewey, 2011), with gene abundance being represented by fragments per kilobase of exon model per million mapped fragments (FPKM). For differential gene expression analysis, we created the count matrix of integer values and the metadata matrix based on the sequencing results (un‐normalized estimated counts). The R package DESeq2 1.4.5 software was used for count normalization and differential gene expression analysis of RNA‐seq data (Love et al., 2014). Differentially expressed genes (DEGs) (Fold change > 1.5 and *Q* value < 0.05) were included for further functional analysis based on GO and KEGG databases. The DEGs of enriched KEGG pathways were used for constructing protein–protein interaction (PPI) network on the STRING online database (http://string‐db.org) with integrated scores > 0.7 (Szklarczyk et al., 2019). The network was constructed and visualized using Cytoscape 3.8.0 software (Shannon, 2003). Quantity of RNA‐seq data output including the size profile of RNAs was listed in Supplementary Table 2 and the details of all the identified genes were listed in Supplementary Table 3.

### 2.9 ApoV uptake by liver macrophages in vivo

Purified apoVs were labelled with fluorescent lipophilic tracer DiR (Invitrogen, USA) according to the manufacturer's instructions, and injected intravenously into C57BL/6 mice. The harvested organs were imaged using the IVIS Spectrum (PerkinElmer, USA) to assess the biodistribution of apoVs. Intensity of fluorescence was quantified with Living Image software (PerkinElmer, USA) (Wiklander et al., 2015).

To verify the uptake of apoVs at the histological level, apoVs were labelled with PKH26 as stated above. Then, the NC suspension and PKH26‐labeled apoVs were injected intravenously into C57BL/6 mice respectively (Liu et al., 2018). At 24 h after injection, the heart, lung, liver, spleen, pancreas and kidney were harvested, fixed in 4% PFA, cryoprotected with 30% sucrose, and embedded in optimal cutting temperature (OCT) compound (Leica, Germany). Then, 15 μm cryosections were prepared (CM1950, Leica, Germany) and counterstained with Hoechst 33342 (Sigma‐Aldrich, USA). For further observation on the uptake of apoVs by macrophages in vivo, immunofluorescent (IF) staining was performed on liver cryosections for macrophage markers F4/80 and CD11b, as stated below. Photographs were taken by a confocal microscope (FV1000, Olympus, Japan) and analyzed using the ImageJ software.

### 2.10 Detection of CRT on the surface of apoVs

For extraction and purification of membrane proteins, apoV sample was pooled, added by PBS supplemented with 1x Protease Inhibitor Cocktail (11697498001, Roche, Germany), and ultrasonized for 5 min in an ice water bath. After centrifugation at 300 g, 4℃ for 10 min, the supernatant was transferred to a new tube and centrifuged at 150,000 g, 4℃ for 1 h. The precipitate was resuspended in 500 μl PBS and centrifuged at 150,000 g, 4℃ for 1 h. The pellet was resuspended in lysis buffer containing SDS L3 and 1x Protease Inhibitor Cocktail, and placed on ice for 5 min. DTT (0281, Amresco, USA) at the final concentration of 10 mM was added and the sample was ultrasonized for 1 min in an ice water bath. After centrifugation at 25,000 g, 4℃ for 10 min, the supernatant was added by 10 mM DTT and placed in a water bath at 56℃ for 1 h, followed by incubation with 55 mM IAM (I6125, Sigma‐Aldrich, USA) for 45 min in the dark. The mixture was then centrifuged at 25,000 g, 4℃ for 15 min and the supernatant was purified membrane protein. The expression of CRT was detected by western blotting using anti‐CRT and anti‐β‐Actin antibodies, as stated below.

For enzyme‐linked immunosorbent assay (ELISA) analysis, the concentration of CRT on apoVs was measured using a commercially available ELISA kit (CUSABIO, China), following the manufacturer's instructions.

For IF staining analysis, apoVs were resuspended in PBS and incubated with anti‐CRT antibody (12238, Cell Signaling Technology, USA; diluted at 1:100) for 1 h at 4℃. Then, apoVs were washed twice with PBS, and incubated with Alexa Fluor 568‐conjugated secondary antibody (A11036, Invitrogen, USA; diluted at 1:100) for 30 min at 4℃. After the incubation, apoVs were washed twice and observed *via* a Axio Observer 5 microscope (Zeiss, Germany). The apoVs stained with only secondary antibody was used as the NC.

For flow cytometric analysis, apoVs underwent primary antibody staining as stated above, followed by incubation with PE‐conjugated secondary antibody (406421, Biolegend, USA; diluted at 1:100), and analyzed on a ACEA NovoCyte flow cytometer (ACEA Biosciences, USA). The apoVs stained with only secondary antibody was used as the NC. Data analysis was performed using the FlowJo 10.0 software (Flow Jo LLC, USA).

### 2.11 Pre‐treatment with trypsin

For trypsin pre‐treatment, purified apoVs were resuspended in trypsin (0.25%) at 2 μg/μl and incubated at 37℃ for 1 h, as previously reported (Figliol, 2020; Togliatto et al., 2018). After washing with PBS, the apoVs were pelleted by centrifugation and used for the indicated assays.

### 2.12 Blocking with neutralizing antibody

To block CRT on the apoV surface, purified apoVs were resuspended in PBS at 2 μg/μl and incubated with CRT blocking antibody (NB600‐101, Novus Biologicals, USA; diluted at 1:100) at 37℃ for 1 h, as previously reported (Chen et al., 2018). Then, the apoVs were washed with PBS to get rid of the non‐bound free antibodies and used for the indicated assays.

### 2.13 Small interferon RNA (siRNA) knockdown

MSCs were transfected with siRNA‐negative control (si‐NC) or siRNA‐*CRT* (si‐*CRT*) (RiboBio, China) using a transfection kit (C10511‐1, RiboBio, China), following the manufacturer's instructions. Transfection efficiency was measured 48 h post‐transfection *via* quantitative real time polymerase chain reaction (qRT‐PCR) and 72 h post‐transfection *via* western blotting. The MSCs transfected with siRNA for 72 h at final concentration of 100 nM were used for apoV isolation.

### 2.14 Magnetic enrichment of apoVs

Enrichment of CRT‐positive apoV subpopulations was performed according to the procedure of magnetic‐activated cell sorting (MACS) with some adjustment (Barnes et al., 2004). Briefly, apoVs were incubated with anti‐CRT antibody and PE‐conjugated secondary antibody, as stated above, followed by incubation with anti‐PE MicroBeads (130‐048‐801, Miltenyi Biotec, Germany). The mixture was washed with PBS and passed through a column within a magnetic field. The flow‐through fraction was defined as the negative fraction and the positive fraction remaining in the column were recovered by elution after turning off the magnetic field. The expression of CRT within both fractions was detected *via* fluorescent observation and flow cytometric analysis, as stated above.

### 2.15 Liver IF staining

IF staining was performed according to previous studies (Chen et al., 2020; Dou et al., 2020). Briefly, air‐dried cryosections of liver specimens were permeabilized by 0.3% Triton X‐100 (Sigma‐Aldrich, USA) for 20 min, blocked in 5% serum (Sigma‐Aldrich, USA) for 30 min, and probed with the primary antibodies overnight at 4℃. The primary antibodies used were as follows: anti‐F4/80 (ab6640, Abcam, UK; diluted at 1:200), anti‐CD11b (101201, Biolegend, USA; diluted at 1:100), anti‐tumour necrosis factor‐alpha (TNF‐α) (NBP1‐19532, Novus Biologicals, USA; diluted at 1:100), anti‐CD206 (MCA2235, Bio‐Rad Laboratories, USA; diluted at 1:100) and anti‐chemokine (C‐C motif) ligand 2 (CCL2) (14‐7096‐81, Invitrogen, USA; diluted at 1:100). After primary antibody incubation, sections were washed for 3 times with PBS and incubated with appropriate Alexa Fluor‐conjugated secondary antibodies (Molecular Probes, USA) for 1 h at room temperature. Then, sections were washed for 3 times with PBS and nuclei were counterstained with Hoechst 33342 (Sigma‐Aldrich, USA). Photographs were taken by a confocal microscope (FV1000, Olympus, Japan) and analyzed using the ImageJ software.

### 2.16 Flow cytometric analysis of liver and blood

Liver perfusion was performed as described previously with slight modification (Liu et al., 2017; Mederacke et al., 2015). Briefly, mice were anesthetized and livers were perfused *via* portal vein catheterization successively with pre‐warmed Hank's balanced salt solution (HBSS), HBSS containing 1 mM ethylene glycolbis(aminoethylether)‐tetra‐acetic acid (EGTA), and HBSS containing 40 μg/ml Liberase (Sigma‐Aldrich, USA) and 5 mM CaCl_2_. After perfusion, livers were removed, gently disassociated with forceps in Dulbecco's modified Eagle's medium (DMEM) (Gibco, USA) containing 5% FBS, and filtered through a 70 μm cell strainer. The nonparenchymal cells were isolated *via* centrifuging at 50 g for 3 min followed by 350 g for 6 min at 4℃, and subjected to red blood cell lysis. Additionally, whole blood was collected from mice, subjected to red blood cell lysis, washed twice with PBS and filtered through a 70 μm cell strainer to obtain single cell suspension. Cells were incubated with anti‐mouse CD16/CD32 mAb (BD Biosciences, USA) for 5 min at 4℃ and then with fluorophore‐conjugated antibodies or isotype controls for an additional 30 min. The antibodies used were as follows: PE‐conjugated anti‐CD45 (12‐0451‐83, eBioscience, USA; diluted at 1:400), Alexa Fluor 488‐conjugated anti‐F4/80 (123120, BioLegend, USA; diluted at 1:400), APC/Cy7‐conjugated anti‐CD11b (101226, BioLegend, USA; diluted at 1:100) and Brilliant Violet 421‐conjugated Ly6G (127627, BioLegend, USA; diluted at 1:200). After incubation with antibodies, cells were washed twice and analyzed on a CytoFLEX flow cytometer (Beckman Coulter, USA). Dead cells were excluded by 4,6‐diamidino‐2‐phenylindole dihydrochloride (DAPI) (BD Biosciences, USA) staining. Data analysis was performed using the FlowJo 10.0 software (Flow Jo LLC, USA).

### 2.17 Adoptive cell transfer

Monocytes were isolated from bone marrow with EasySep mouse monocytes enrichment kit (STEMCELL tech, Canada) according to the instructions. Cells were suspended in PBS, filtered through a 70 μm cell strainer and labelled with PKH67 (Sigma‐Aldrich, USA). Then, 1×10^6^ cells in 200 μl PBS were injected *via* caudal vein into recipient mice as previously described (Li et al., 2016; Li et al., 2015). Two days after injection, in vivo cell tracking was performed by liver flow cytometric analysis.

### 2.18 RNA isolation and qRT‐PCR

Total RNA was extracted from liver tissues or cells using Trizol Reagent (Takara, Japan), and complementary DNA (cDNA) was generated using a PrimeScriptTM RT Reagent Kit (Takara, Japan). Then, qRT‐PCR was performed with a SYBR Premix Ex Taq II Kit (Takara, Japan) by a Real‐Time System (CFX96, Bio‐Rad, USA). Quantification was performed by using β‐actin (*Actb or ACTB*) as the internal control and calculating the relative expression level of each gene with the 2^–ΔΔCT^ method as previously described (Zheng et al., 2018). Values were expressed as fold changes. All the primer sequences were listed in Supplementary Table 4.

### 2.19 ELISA of cytokines

For serum examination, mice were anaesthetized and the whole peripheral blood were obtained from the retro‐orbital venous plexus. Then, serum was isolated *via* centrifuging at 3,000 rpm for 10 min followed by 12,000 rpm for 10 min as previously described (Zheng et al., 2018). For tissue lysate examination, liver tissues were rinsed with PBS and homogenized in ice cold lysis buffer with protease inhibitors, followed by centrifuging at 2,000 rpm for 5 min, as instructed by the manufacture. For media examination, culture supernatants from BMDMs were collected and cleared by centrifugation to remove cell debris (Postat et al., 2018). The concentrations of TNF‐α, interleukin‐10 (IL‐10) and CCL2 in serum and media as well as CCL2 in liver lysates were detected using murine ELISA kits following the manufacturer's instructions (R&D Systems, USA).

### 2.20 Glucose tolerance tests (GTTs) and insulin tolerance tests (ITTs)

For GTTs, mice were fasted for 16 h and intraperitoneally injected with dextrose (2 g/kg body weight). Blood glucose levels were measured with blood drawn from the tail using a glucometer (Roche, Germany) at 0, 15, 30, 60, 90 and 120 min after dextrose administration (Ying et al., 2017).

For ITTs, mice were fasted for 6 h and intraperitoneally injected with recombinant human insulin (1 IU/kg body weight). Blood glucose levels were measured at 0, 15, 30, 60, 90 and 120 min after insulin administration (Ying et al., 2017).

### 2.21 Hepatic insulin signalling

Liver insulin signalling was evaluated by measuring insulin‐stimulated AKT phosphorylation as previously described (Okin & Medzhitov, 2016; Ying et al., 2017). Briefly, mice were fasted for 8 h and then injected intraperitoneally with recombinant human insulin (1 IU/kg body weight). Mice were sacrificed at 15 min after injection and liver tissues were collected and snap frozen with liquid nitrogen. The phosphorylation of AKT was determined by western blotting with anti‐phosphorylated AKT (Ser473) and anti‐AKT antibodies, as stated below.

### 2.22 Histological analysis

At sacrifice, liver tissues were dissected, fixed in 4% PFA, and underwent dehydration with graded ethanol. Then, 5 μm paraffin‐embedded liver sections were prepared (RM2125, Leica, Germany) and stained with haematoxylin and eosin (H&E) (Li et al., 2016). For ORO staining, dissected liver tissues were flash‐frozen with liquid nitrogen and imbedded in OCT compound (Leica, Germany). Then, 10 μm cryo‐sections were prepared (CM1950, Leica, Germany), immersed in ORO working solution for 5 min and rinsed with H_2_O. Sections were counterstained with Mayer's haematoxylin and mounted (Mehlem et al., 2013). The stained sections were photographed with a microscope (M205FA, Leica, Germany).

### 2.23 Lipid contents measurement

For tissue lysate examination, at sacrifice, liver tissues were dissected and homogenized in ethyl alcohol buffer on ice according to the manufacturer's instructions. The triglyceride (TG) and total cholesterol (TC) levels in the liver were measured by the commercial kits according to the protocols (Nanjing Jiancheng Biology Engineering Institute, China).

### 2.24 Western blotting

Western blotting was performed according to previously studies (Chen et al., 2020; Sui et al., 2017). Whole lysates of tissues, cells or apoVs were prepared using the Lysis Buffer (Beyotime, China) supplemented with protease and phosphatase inhibitors (Roche, Germany). Proteins were extracted and the protein concentration was quantified using a BCA Protein Assay Kit (TIANGEN, China). Equal amounts of protein samples were loaded onto SDS–PAGE gels and transferred to polyvinylidene fluoride (PVDF) membranes (Millipore, USA) which were blocked with 5% bovine serum albumin (BSA) (Sigma‐Aldrich, USA) in TBS for 2 h at room temperature. Then, the membranes were incubated overnight at 4℃ with the following primary antibodies: anti‐Caspase‐3 (9662, Cell Signaling Technology, USA; diluted at 1:1000), anti‐CRT (12238, Cell Signaling Technology, USA; diluted at 1:1000), anti‐phosphorylated AKT (Ser473) (4060, Cell Signaling Technology, USA; diluted at 1:1000), anti‐AKT (9272, Cell Signaling Technology, USA; diluted at 1:1000) and anti‐β‐Actin antibodies (CW0096, CWBio, China; diluted at 1:2000). After washing with TBS containing 0.1% Tween‐20, the membranes were incubated with peroxidase‐conjugated secondary antibodies (Jackson ImmunoResearch, USA) for 1 h at room temperature. The protein bands were visualized using an enhanced chemiluminescence kit (Amersham Biosciences, USA) and detected by a gel imaging system (4600, Tanon, China).

### 2.25 Statistical analysis

All the data were presented as mean ± standard deviation (SD). Statistical and graph analyses were performed using GraphPad Prism 8.0 (GraphPad Software, USA). For two group comparisons, significance was assessed by Student's *t* test (two‐tailed) or Student's *t* test with Welch correction (two‐tailed). For multiple group comparisons, significance was assessed by One‐way ANOVA with Tukey's post hoc test, Welch's ANOVA with Tamhane's T2 post hoc test or Kruskal‐Wallis H test. Values of *P* < 0.05 were considered statistically significant.

## 3 RESULTS

### 3.1 MSC‐derived apoVs are enriched with a variety of functional proteins

**FIGURE 1:**
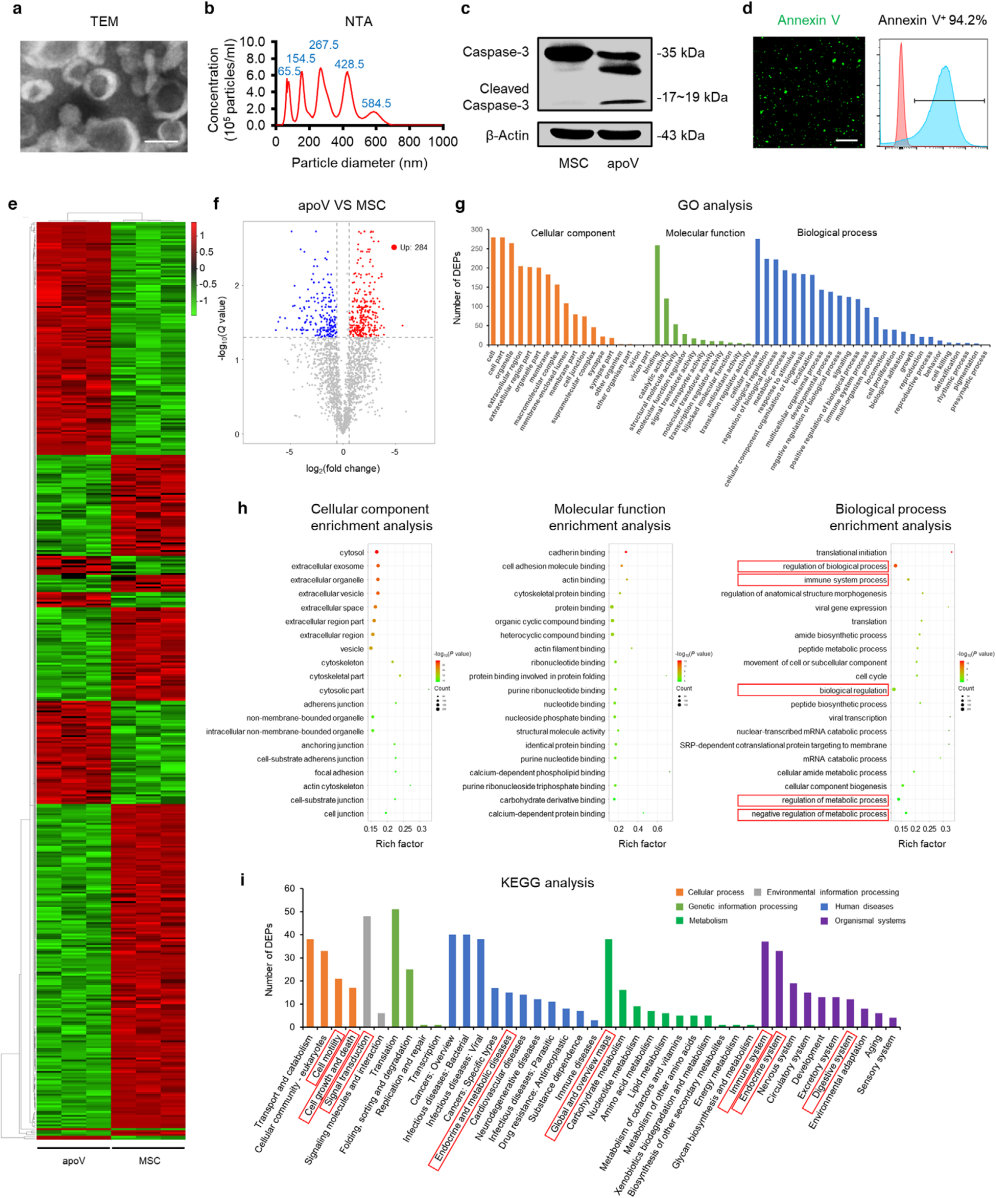
Characterization and proteomic analysis of mesenchymal stem cell (MSC)‐derived apoptotic vesicles (apoVs). (a) Representative transmission electron microscope (TEM) image showing the morphology of apoVs. Scale bar, 125 nm. (b) Nanoparticle tracking analysis (NTA) showing the size distribution of apoVs. (c) Western blotting analysis showing the presence of Caspase‐3/Cleaved Caspase‐3 in MSCs and apoVs. (d) Representative confocal microscopy images and flow cytometric analysis of Annexin V (green) staining in apoVs. Scale bars, 10 μm. (e) Hierarchical clustering of differentially expressed proteins (DEPs) (Fold change > 1.5 and *Q* value < 0.05) between MSCs and apoVs, with protein abundance being Z‐score normalized. Rows represent proteins and columns represent individual replicates. (f) Volcano plot showing significantly upregulated (red dots) and downregulated (blue dots) proteins in apoVs, compared to MSCs. (g) Gene ontology (GO) analysis of significantly upregulated proteins in apoVs, categorized into ‘Cellular component’, ‘Molecular function’ and ‘Biological process’. (h) GO enrichment analysis of significantly upregulated proteins in apoVs. The top twenty enriched terms of the three categories in (g) were respectively presented as bubble charts. The Y‐axis represents GO terms and the X‐axis represents rich factor. The colour of the bubble represents enrichment significance and the size of the bubble represents number of upregulated proteins. (i) Kyoto Encyclopedia of Genes and Genomes (KEGG) pathway analysis of significantly upregulated proteins in apoVs

We characterized MSCs used in this study. Consistent with the International Society of Cell Therapy (ISCT) criteria (Dominici et al., 2006), MSCs highly expressed CD105, CD73 and CD90 while they were negative for CD45, CD34 and CD11b (Supplementary Figure 1(A)). Also, MSCs showed spindle shape and were able to generate single colony clusters (Supplementary Figure 1(B)). While the MSCs used for apoV isolation were undifferentiated, MSCs possess osteogenic and adipogenic differentiation potential after being induced in osteogenic or adipogenic medium, as shown by alizarin red S staining and ORO staining (Supplementary Figure 1(B)). For the induction of apoptosis, MSCs were washed with PBS and then treated with STS, which showed significant morphological alteration and positive TUNEL staining (Supplementary Figure 1(C)). We isolated apoVs from apoptotic MSCs using the optimized gradient centrifugation protocol (Liu et al., 2020) (Supplementary Figure 2(A)) and characterized apoVs in terms of morphology (Figure 1(a), Supplementary Figure 2(B, C)), size distribution (Figure 1(b), Supplementary Figure 2(D)), presence of apoptosis‐associated markers (Figure 1(c)), and exposure of phosphatidylserine (PtdSer) (Figure 1(d)).

The molecular composition of EVs is a key focus in the field, as they carry a wide variety of functional proteins (Choi et al., 2020; Hoshino et al., 2020). To identify the specific proteomic features of MSC‐derived apoVs, we prepared proteins of apoVs and parental MSCs, and performed LC‐MS/MS analysis. A total of 2873 proteins were identified (Supplementary table 1), while 481 were DEPs (Figure 1(e)), within which 284 were significantly upregulated in apoVs (Figure 1(f)). We then focused on these enriched proteins and carried out functional analysis. We found that several key apoptotic markers have been significantly upregulated in apoVs validating their identity, such as the apoptosis regulator BAX (Walensky, 2019; Westphal et al., 2014), gelsolin (GSN) (Kothakota, 1997; Springer et al., 1999), mitogen‐activated protein kinase kinase kinase kinase 4 (MAP4K4) (Fiedler et al., 2019) and reticulon‐4 (RTN4) (Chen et al., 2010), the details of which have been listed in Supplementary table 5. As to the functional annotations based on the GO database, the upregulated proteins were included into multiple terms within the three domains, namely ‘Cellular component’, ‘Molecular function’ and ‘Biological process’, suggesting load of various functional proteins in apoVs (Figure 1(g)). Enrichment analysis revealed that apoVs had increased expression of proteins with regulatory functions, including ‘regulation of biological process’, ‘immune system process’, ‘biological regulation’, ‘regulation of metabolic process’ and ‘negative regulation of metabolic process’ (Figure 1(h)). Furthermore, we explored the pathways that were involved in the upregulated proteins *via* KEGG pathway analysis. Results showed that these proteins were associated with ‘Cell motility’ and ‘Cell growth and death’ within the ‘Cellular process’ domain, ‘Signal transduction’ within the ‘Environmental information processing’ domain, ‘Endocrine and metabolic diseases’ within the ‘Human diseases’ domain, ‘Global and overview maps’ within the ‘Metabolism’ domain, and ‘Immune system’, ‘Endocrine system’ and ‘Digestive system’ within the ‘Organismal systems’ domain (Figure 1(i)). These findings suggest that apoVs are enriched with a set of functional proteins that are highly related to cellular behaviour, signalling transduction, as well as regulation of immune and metabolism. In specific, we demonstrated that several proteins with the potential to induce macrophage anti‐inflammatory (M2) polarization were enriched in apoVs, including alpha‐crystallin B chain (CRYAB) (Ousman et al., 2007; Zhang et al., 2015), cAMP‐dependent protein kinase type II‐alpha regulatory subunit (PRKAR2A) (Kong et al., 2016), receptor of activated protein C kinase 1 (RACK1) (Dan et al., 2020) and vasodilator‐stimulated phosphoprotein (VASP) (Laban et al., 2018; Lee et al., 2015), which might mediate the modulatory effects of apoVs. A detailed list of these proteins can be found in Supplementary table 6.

### 3.2 Efferocytosis of MSC‐derived apoVs induces transcriptional reprogramming of macrophages

**FIGURE 2:**
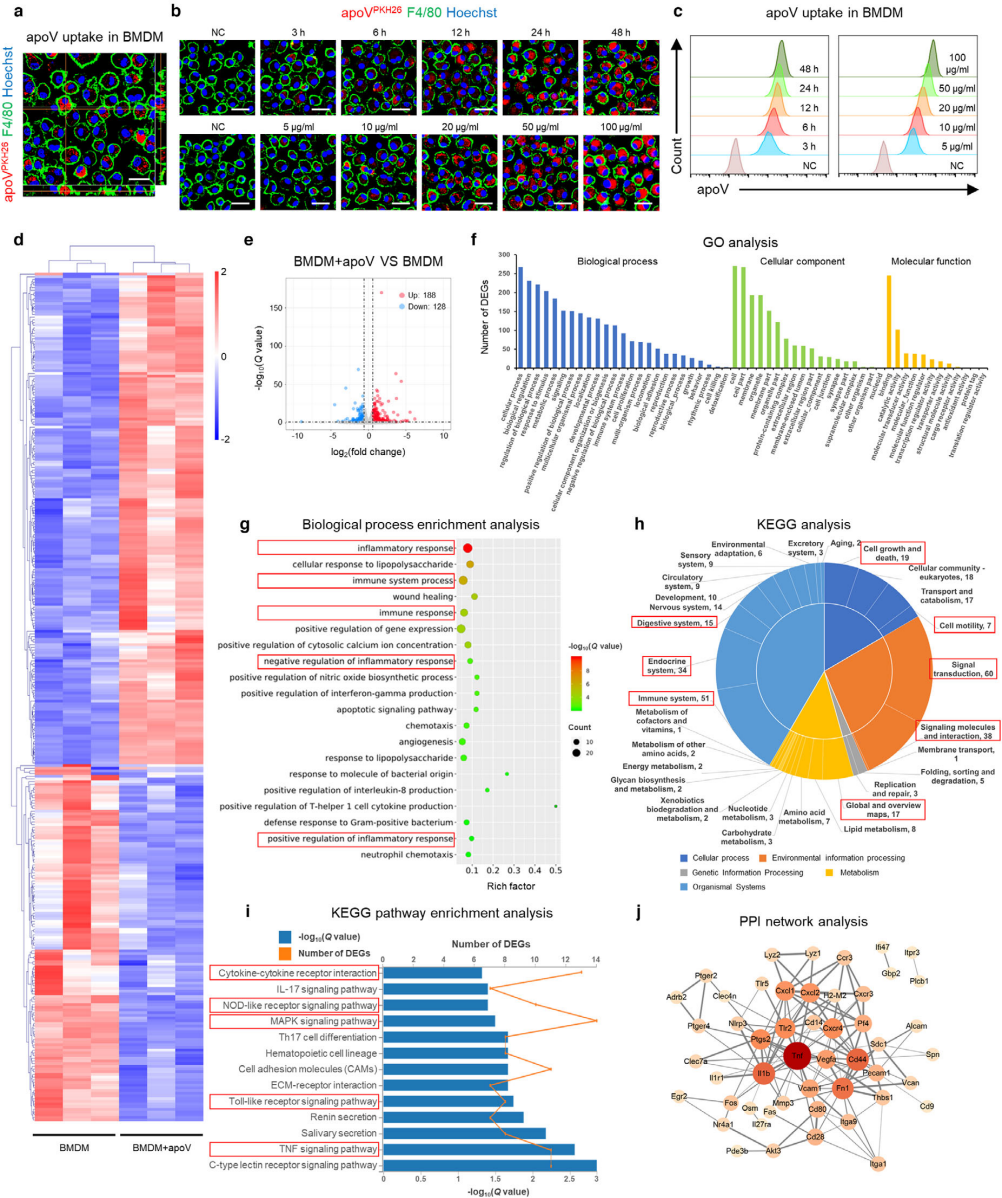
Efferocytosis of MSC‐derived apoVs induces transcriptional reprogramming in bone marrow‐derived macrophages (BMDMs). (a) Representative confocal orthogonal view showing uptake of PKH26‐labeded apoVs (red) by BMDMs (green), counterstained by Hoechst (blue). Scale bar, 20 μm. (b) Representative confocal microscopy images showing time‐dependent and concentration‐dependent uptake of PKH26‐labeded apoVs (red) by BMDMs (green), counterstained by Hoechst (blue). After removal of unbound PKH, the stained apoVs were resuspended in PBS and underwent centrifugation, after which the supernatant was used as the negative control (NC) and added to BMDMs. Scale bars, 25 μm. (c) Flow cytometric analysis of time‐dependent and concentration‐dependent uptake of PKH67‐labeded apoVs by macrophages. NC was prepared as stated above. (d) Hierarchical clustering of differentially expressed genes (DEGs) (Fold change > 1.5 and *Q* value < 0.05) between BMDMs and apoV‐treated BMDMs (BMDM+apoV), with gene abundance being Z‐score normalized. Rows represent genes and columns represent individual replicates. (e) Volcano plot showing DEGs in BMDM+apoV compared to BMDMs. The blue and red dots indicate downregulated and upregulated genes, respectively. (f) Gene ontology (GO) analysis of the DEGs in (e), categorized into ‘Cellular component’, ‘Molecular function’ and ‘Biological process’. (g) ‘Biological process’ enrichment analysis of the DEGs in (e). The top twenty enriched terms were presented as a bubble chart. The Y‐axis represents GO terms and the X‐axis represents rich factor. The colour of the bubble represents enrichment significance and the size of the bubble represents the number of DEGs. (h) Kyoto Encyclopedia of Genes and Genomes (KEGG) pathway analysis of the DEGs in (e). The number of genes annotated into different categories is shown. (i) KEGG pathway enrichment analysis of the DEGs in (e). The enriched KEGG pathways were presented as a bar chart. The Y‐axis represents KEGG pathways and the X‐axes represents number of DEGs (top) and enrichment significance (low), respectively. (j) Protein‐protein interaction (PPI) network analysis of the DEGs belonging to the enriched KEGG pathways in (i)

Considering that macrophages are the major professional phagocytes responsible for efferocytosis (Boada‐Romero et al., 2020; Doran et al., 2020; Morioka et al., 2019), we aimed to investigate whether efferocytosis of apoVs triggers functional responses in macrophages. BMDMs were characterized *via* flow cytometric analysis (Supplementary Figure 2(E)) and then PKH26‐labeled apoVs, at various time points and concentrations, were added to cultured BMDMs. Internalization of apoVs were verified by confocal microscopy observation (Supplementary Figure 2(E) and Figure 2(a)), showing a time‐ and concentration‐dependent manner (Figure 2(b, c)) (Dou et al., 2020). A negative control was used to prove that unbound PKH has been removed (Figure 2(b, c)).

To elucidate whether efferocytosis of MSC‐derived apoVs influences the gene expression profiles of BMDMs, we performed RNA‐seq analysis on apoV‐treated BMDMs and control BMDMs. In total, we identified 316 DEGs induced after apoV engulfment (Figure 2(d)), consisting of 188 upregulated genes and 128 downregulated genes (Figure 2(e)). With regard to the functional analysis based on GO database, the DEGs were annotated to multiple terms within the three GO categories ‘Biological process’, ‘Cellular component’ and ‘Molecular function’, implying distinct transcriptional changes induced by internalization of apoVs (Figure 2(f)). Specifically, concerning the ‘Biological process’ category, there was an obvious enrichment of DEGs linked to immunity and inflammation, including ‘inflammatory response’, ‘immune system process’, ‘immune response’, ‘negative regulation of inflammatory response’ and ‘positive regulation of inflammatory response’ (Figure 2(g)). KEGG pathway analysis also revealed that apoVs altered expression of genes associated with cellular behaviour, signalling transduction as well as regulation of immunity and metabolism, such as ‘Cell motility’, ‘Cell growth and death’, ‘Signal transduction’, ‘Signalling molecules and interaction’, ‘Global and overview maps’ of metabolism, ‘Immune system’, ‘Endocrine system’ and ‘Digestive system’ (Figure 2(h)). Further enrichment analysis showed that multiple inflammatory‐related pathways, *e.g*. ‘TNF signalling pathway’, ‘Toll‐like receptor signalling pathway’, ‘MAPK signalling pathway’, ‘NOD‐like receptor signalling pathway’ and ‘Cytokine‐cytokine receptor interaction’, were highly enriched for these DEGs (Figure 2(i)). PPI analysis was performed to show an elaborate network of proteins with functional interactions (Figure 2(j)). In specific, TNF‐α played a central role in the network (Figure 2(j)). These data indicate that efferocytosis of MSC‐derived apoVs leads to transcriptional reprogramming of macrophages in vitro.

### 3.3 Efferocytosis of MSC‐derived apoVs alleviates macrophage infiltration and activation in T2D liver

**FIGURE 3:**
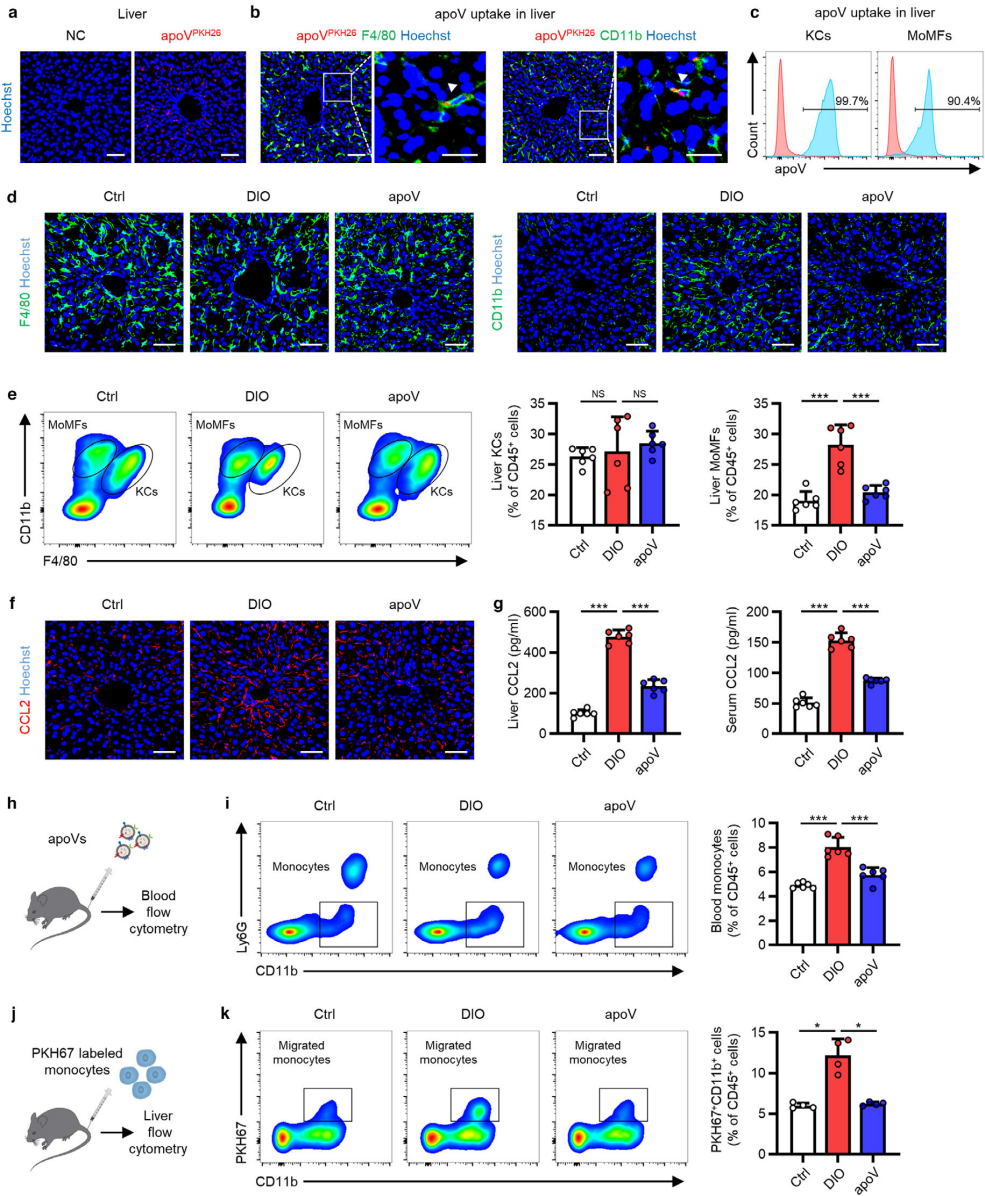
Efferocytosis of MSC‐derived apoVs by liver macrophages alleviates macrophage infiltration in the type 2 diabetes (T2D) liver. (a) Representative confocal microscopy images showing distribution of PKH26‐labeled apoVs (red) in the liver, counterstained by Hoechst (blue). After removal of unbound PKH, the stained apoVs were resuspended in PBS and underwent centrifugation, after which the supernatant was used as the negative control (NC) and injected. Scale bars, 50 μm. (b) Representative confocal microscopy images showing uptake of apoVs (red) by macrophages (green) in the liver, counterstained by Hoechst (blue). Scale bars, 50 μm in low magnification images and 25 μm in high magnification images. (c) Flow cytometric analysis showing the uptake of apoVs by macrophages in the liver. KCs, Kupffer cells; MoMFs, monocyte‐derived macrophages. (d) Representative immunofluorescent (IF) staining images of F4/80 (green) and CD11b (green) in the liver, counterstained by Hoechst (blue). Ctrl, control mice; DIO, mice with diet‐induced obesity; apoV, DIO mice treated by apoVs. Scale bars, 50 μm. (e) Flow cytometric analysis and the corresponding quantification of the percentages of KCs and MoMFs in hepatic CD45^+^ cells. *N* = 6 per group. (f) Representative IF staining images of chemokine (C‐C motif) ligand 2 (CCL2) (red) in the liver, counterstained by Hoechst (blue). Scale bars, 50 μm. (g) Enzyme‐linked immunosorbent assay (ELISA) analysis of CCL2 in liver lysate and serum. *N* = 6 per group. (h) Schematic diagram showing systemic injection of apoVs into mice which undergo blood flow cytometric analysis. (i) Flow cytometric analysis and the corresponding quantification of the percentages of monocytes in the peripheral blood CD45^+^ cells. *N* = 6 per group. (j) Schematic diagram showing injection and in vivo tracking of PKH67‐labeled bone marrow monocytes. (k) Flow cytometric analysis and the corresponding quantification of the percentages of PKH67‐labeled monocytes migrating to the liver. *N* = 4 per group. Data are presented as mean ± standard deviation (SD). Statistical analyses are performed by One‐way ANOVA with Tukey's post hoc test or Welch's ANOVA with Tamhane's T2 post hoc test. *, *P* < 0.05; ***, *P* < 0.001; NS, *P* > 0.05

It has been well established that chronic inflammation characterized by infiltration and activation of macrophages is a key mechanism for metabolic diseases, including T2D (Castegna et al., 2020; Mcnelis & Olefsky, 2014). As a major metabolic organ, the liver comprises two macrophage populations, Kupffer cells (KCs), the resident specialized hepatic macrophage, and monocyte‐derived macrophages (MoMFs), the recruited macrophages arising from circulating monocytes. Under overnutrition condition, activated pro‐inflammatory KCs initiate the inflammation and induce the infiltration of MoMFs which display highly pro‐inflammatory phenotype and lead to insulin resistance as well as hepatic steatosis (Kazankov et al., 2019; Lee et al., 2018; Morinaga et al., 2015). In this study, we intended to decipher the in vivo fate of systemically infused apoVs and unveil their effects on HFD‐induced T2D model. We analyzed the biodistribution of systemically delivered apoVs in various organs (heart, lung, liver, spleen, pancreas and kidney) and found significant accumulation of apoVs in the liver at 24 h post‐injection (Supplementary Figure 2(F), Figure 3(a)), which was consistent with previous studies showing a centre role of the liver in organismal clearance (Poon et al., 2019; Tsoi et al., 2016). We proceeded to explore whether apoVs were engulfed by liver macrophages. As determined by confocal microscopy and flow cytometric analyses, both KCs and MoMFs engulfed apoVs (Figure 3(b, c)).

Based on the above observation, we proceeded to elucidate whether efferocytosis of MSC‐derived apoVs influences the liver macrophages, in particular the infiltration of MoMFs. Liver sections were stained for F4/80, a marker for KCs (Tosello‐Trampont et al., 2012), and CD11b that preferentially marked MoMFs (Viebahn et al., 2010). Compared to control mice, the number of F4/80^+^ cells showed no significant difference while CD11b^+^ cells were increased in the diet‐induced obesity (DIO)‐induced T2D mice (Figure 3(d)). Flow cytometric analysis further demonstrated that the percentage of KCs (F4/80^high^CD11b^low^) remained unchanged whereas that of MoMFs (CD11b^int^F/80^low^) was significantly upregulated (Figure 3(e)) (Guo et al., 2019; Obstfeld et al., 2010). Importantly, the infiltration of MoMFs was significantly inhibited by efferocytosis of apoVs (Figure 3(d, e)). We detected the changes of CCL2 that is the most important chemokine in mediating MoMFs recruitment (Baeck et al., 2012), and found increased levels of CCL2 in the liver and serum of DIO mice, which was also markedly downregulated by efferocytosis of apoVs (Figure 3(f, g)). Similar changes of peripheral blood monocytes were observed (Figure 3(h, i)). Furthermore, we systemically infused bone marrow monocytes *via* the tail vein and directly detected their migration into the liver using an in vivo tracing approach (Figure 3(j)) (Li et al., 2015, 2016). As expected, there was a substantially lower percentage of migrated monocytes in apoV‐treated DIO mice than vehicle‐treated DIO mice (Figure 3(k)). These data suggest that efferocytosis of MSC‐derived apoVs by liver macrophages mitigates macrophage infiltration in T2D liver.

**FIGURE 4:**
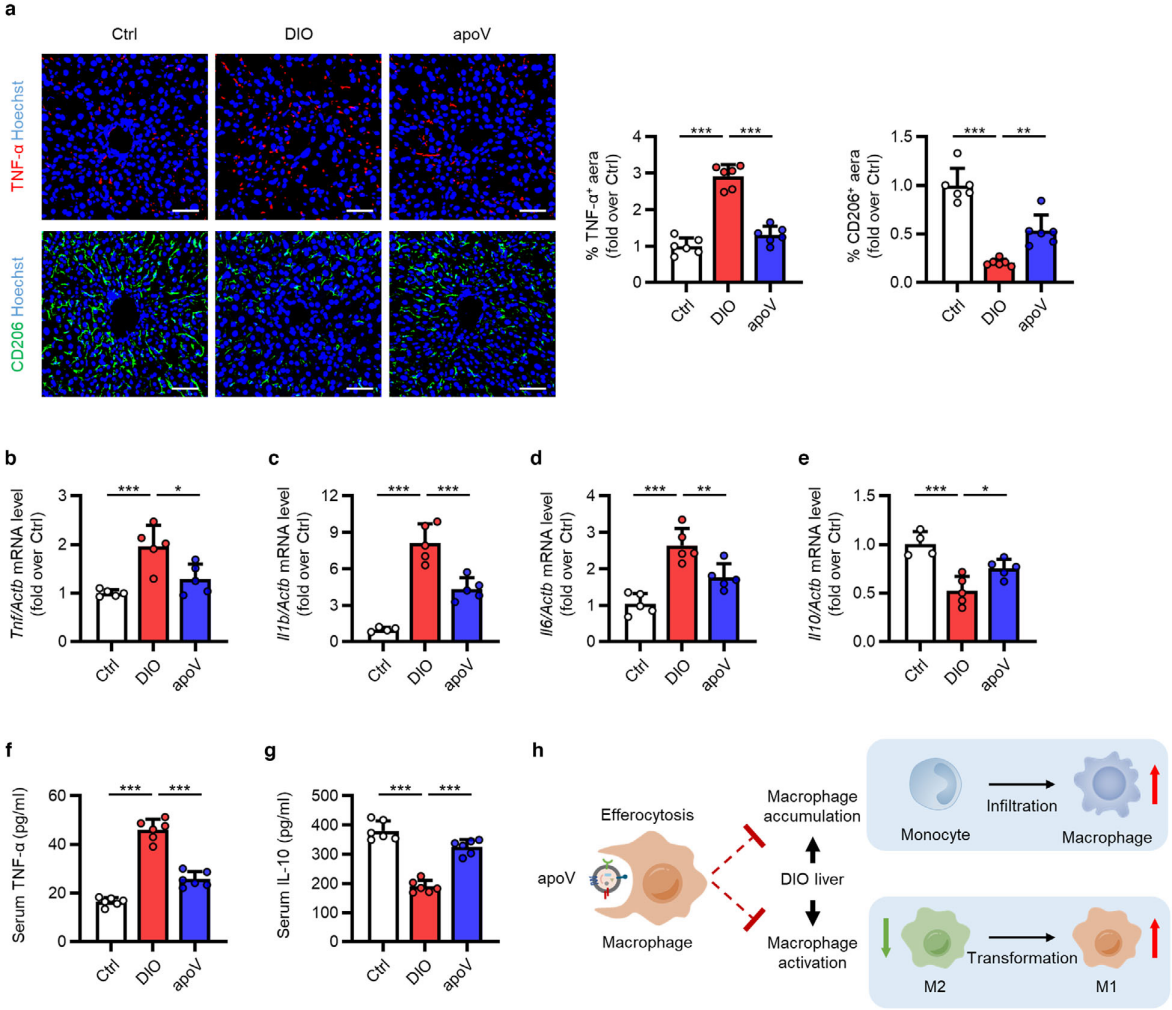
Efferocytosis of MSC‐derived apoVs by liver macrophages alleviates macrophage activation in the T2D liver. (a) Representative immunofluorescent (IF) staining images of tumor necrosis factor‐alpha (TNF‐α) (red) and CD206 (green) in the liver, counterstained by Hoechst (blue), and the corresponding quantification of fold changes over the Ctrl group. Ctrl, control mice; DIO, mice with diet‐induced obesity; apoV, DIO mice treated by apoVs. Scale bars, 50 μm. *N* = 6 per group. (b‐e) Quantitative real time polymerase chain reaction (qRT‐PCR) analysis of the mRNA expression levels of *Tnf* (b), interleukin 1 beta (*Il1b*) (c), interleukin 6 (*Il6*) (d) and interleukin 10 (*Il10*) (e) in the liver, normalized to β‐actin (*Actb*), and quantification of fold changes over the Ctrl group. *N* = 4–5 per group. (f, g) Enzyme‐linked immunosorbent assay (ELISA) analysis of TNF‐α (f) and IL‐10 (g) in serum. *N* = 6 per group. (h) Schematic diagram showing that efferocytosis of apoVs inhibits macrophage accumulation and activation in the liver of DIO mice. M1, pro‐inflammatory macrophages; M2, anti‐inflammatory macrophages. Data are presented as mean ± standard deviation (SD). Statistical analyses are performed by One‐way ANOVA with Tukey's post hoc test. *, *P* < 0.05; **, *P* < 0.01; ***, *P* < 0.001

As stated above, in addition to heterogeneity of developmental origins, hepatic macrophages also display plasticity in polarization, ranging from a pro‐inflammatory phenotype (M1) to an anti‐inflammatory state (M2) (Krenkel & Tacke, 2017; Tacke, 2017). M1 macrophages characterized by the production of pro‐inflammatory cytokines contribute to obesity‐induced insulin resistance and hepatic steatosis while M2 macrophages exert ameliorative effects (Odegaard et al., 2008; Wan et al., 2014). We aimed to explore whether efferocytosis of MSC‐derived apoVs affected liver macrophage polarization. Visualization of macrophage phenotype *via* IF staining demonstrated that M1 macrophages were apparently increased in DIO mice which was blunted after efferocytosis of apoVs (Figure 4(a)). Reduced M2 macrophages were recovered with uptake of apoVs (Figure 4(a)). DIO mice showed a higher mRNA expression levels of M1 markers, including *Tnf*, interleukin 1 beta (*Il1b*) and interleukin 6 (*Il6*), whereas the expression of M2 marker, *Il10*, were downregulated, which was reversed by engulfment of apoVs (Figure 4(b‐e)). The levels of pro‐inflammatory factor, TNF‐α, and anti‐inflammatory factor, IL‐10, in serum showed a consistent change tendency (Figure 4(f, g)). These results indicate that efferocytosis of MSC‐derived apoVs inhibit DIO‐induced macrophage activation in the liver. Collectively, the chronic inflammatory environmental of T2D liver has been improved (Figure 4(h)).

### 3.4 CRT is exposed on the surface of apoVs

**FIGURE 5:**
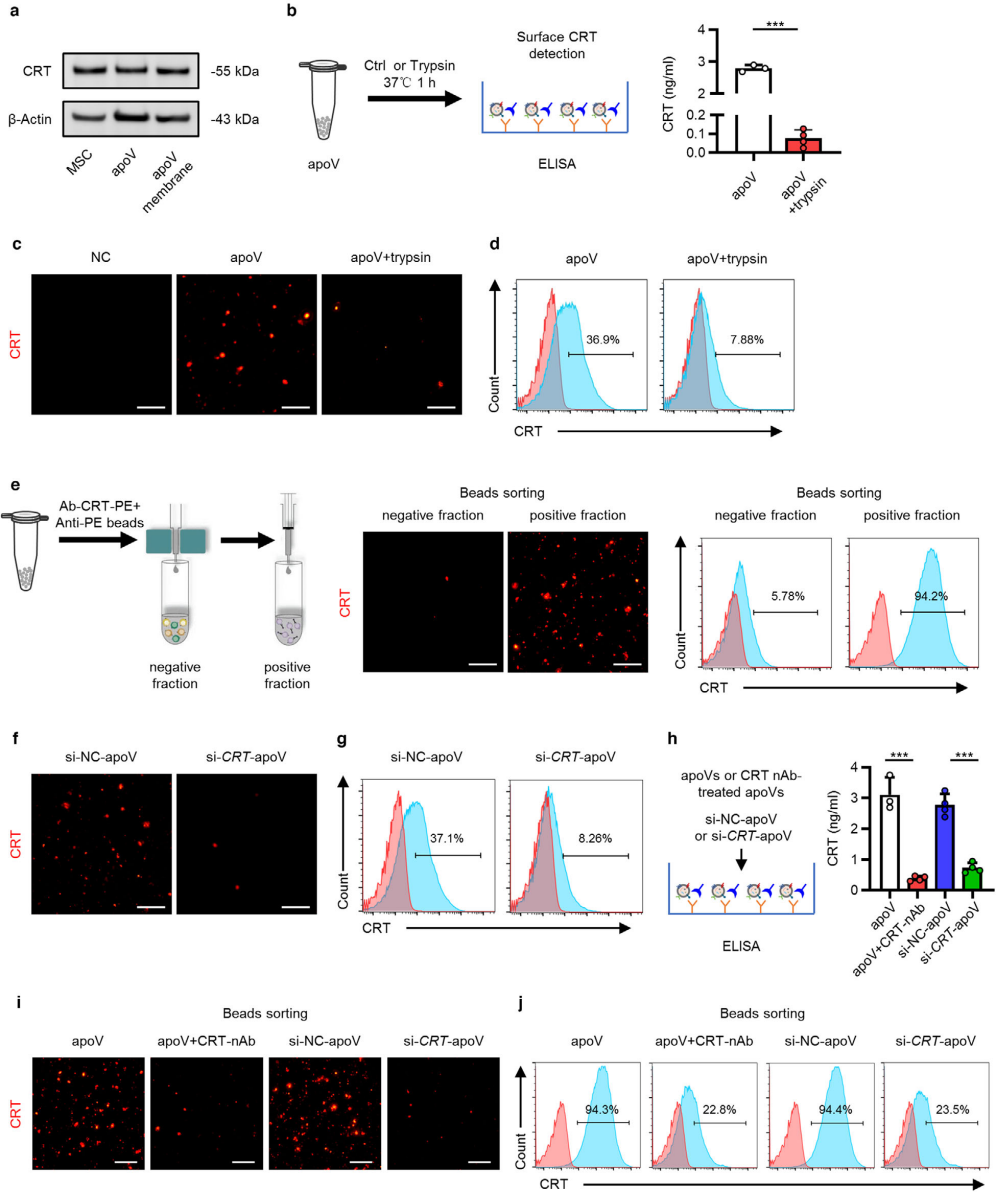
Surface expression of calreticulin (CRT) on apoVs. (a) Western blotting analysis showing the presence of CRT in membrane proteins of apoVs as well as in total proteins of MSCs and apoVs. (b) Schematic diagram showing detection of surface CRT *via* enzyme‐linked immunosorbent assay (ELISA) analysis and quantification of the concentration of CRT. apoV+trypsin, apoVs pre‐treated with trypsin at 37℃ for 1 h. *N* = 3–4 per group. (c) Representative immunofluorescent (IF) staining images of CRT (red) on apoVs. ApoVs incubated with only secondary antibody was used as the negative control (NC). Scale bars, 30 μm. (d) Flow cytometric analysis of CRT on the surface of apoVs. (e) Enrichment of CRT‐positive apoV subpopulations *via* magnetic beads sorting. Left: schematic diagram; Medium: fluorescent images of CRT (red); Right: flow cytometric analysis of CRT. Scale bars, 30 μm. (f) Representative IF staining images of CRT (red). si‐NC‐apoV, apoVs derived from MSCs treated by siRNA‐NC; si‐*CRT*‐apoV, apoVs derived from MSCs treated by siRNA‐*CRT*. Scale bars, 30 μm. (g) Flow cytometric analysis of CRT on the surface of apoVs. (h) ELISA analysis of CRT on the surface of apoVs. apoV+CRT‐nAb, apoVs pre‐treated with CRT neutralizing antibody at 37℃ for 1 h. *N* = 3–4 per group. (i) Representative fluorescent images of CRT (red) after magnetic beads sorting of CRT‐positive apoV subpopulations. Scale bars, 30 μm. (j) Flow cytometric analysis of CRT on the surface of apoVs after magnetic beads sorting of CRT‐positive apoV subpopulations. Data are presented as mean ± standard deviation (SD). Statistical analyses are performed by Student's *t* test (two‐tailed). ***, *P* < 0.001

Efferocytosis is a multistep process tightly regulated by highly conserved mechanisms and signalling pathways (Boada‐Romero et al., 2020; Fond & Ravichandran, 2016). While the process of apoptotic cell efferocytosis has been relatively well‐established, the molecular mechanism for apoVs remains largely unknown. Notably, CRT is a conserved endoplasmic reticulum (ER) protein that has been recognized to be rapidly exposed on cell surface during apoptosis and serve as an important ‘eat‐me’ signal to mediate efferocytosis (Gardai et al., 2005; Kojima et al., 2014; Païdassi et al., 2011; Park et al., 2008). In the light of this knowledge, we intended to explore whether CRT was exposed on the surface of apoVs and mediated the efferocytosis of apoVs. We isolated membrane proteins of apoVs and verified the expression of CRT through western blotting analysis (Figure 5(a)). ELISA assay showed that digestion of surface proteins *via* trypsin pre‐treatment downregulated the expression of CRT (Figure 5(b)). Further examination of intact apoVs and trypsin pre‐treated apoVs through IF staining and flow cytometric analysis proved that CRT was anchored to the membrane of apoVs (Figure 5(c, d)). We used magnetic beads to separate CRT‐positive and CRT‐negative apoVs. The presence of CRT in the separated positive and negative fractions was optically confirmed by IF staining and flow cytometric analysis. As expected, the positive fluorescent signal was high in the positive fraction while only a very low fluorescent signal was detected in the negative fraction (Figure 5(e)). siRNA technology was harnessed to abrogate CRT expression in apoVs. In specific, we transfected MSCs with si‐*CRT* and its NC, and confirmed the down‐regulation of *CRT* gene and CRT protein in apoVs (Supplementary Figure 3(A, B)). We further verified that CRT protein exposed on the surface of apoVs was reduced by si‐*CRT* (Figure 5(f, g)). In order to block the CRT on the surface of apoVs, we pre‐treated apoVs with a CRT neutralizing antibody. ELISA analysis confirmed that CRT levels decreased dramatically after CRT blockade and siRNA‐mediated downregulation (Figure 5(h)). Moreover, the positive fluorescent signal of CRT positive fraction sorted *via* magnetic beads significantly decreased in CRT neutralizing antibody pre‐treated apoVs and si‐*CRT*‐apoVs, suggesting less capture of CRT‐positive apoVs in beads sorting (Figure 5(i, j)). Taken together, these data indicate that CRT is exposed on the surface of apoVs which might mediate the efferocytosis of apoVs.

### 3.5 CRT mediates efferocytosis of MSC‐derived apoVs to modulate T2D macrophage in vitro

**FIGURE 6:**
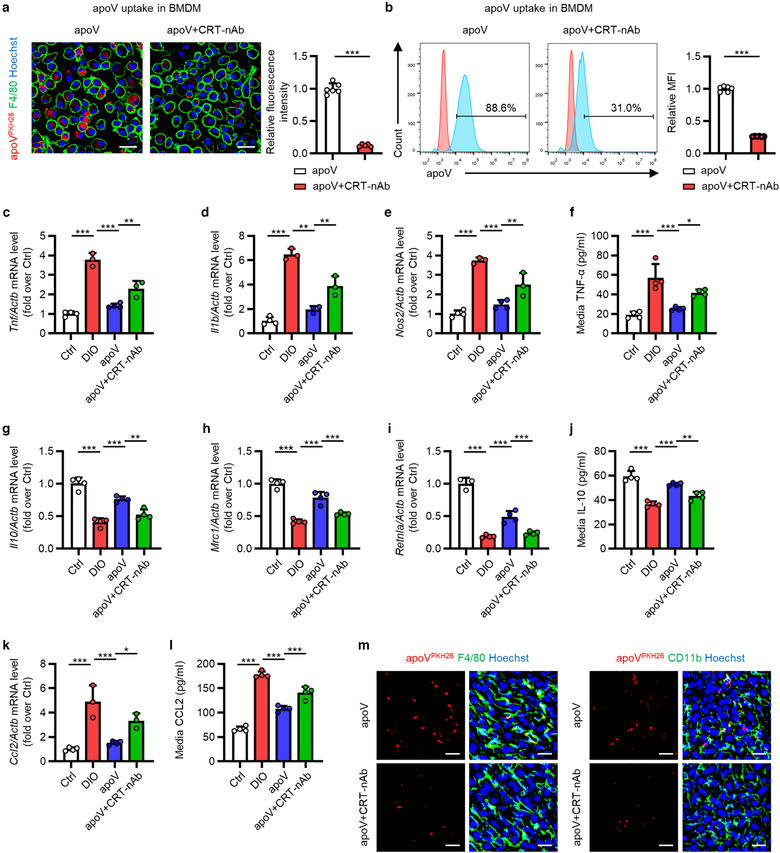
CRT blockade inhibits functional efferocytosis of MSC‐derived apoVs by T2D macrophages in vitro. (a) Representative confocal microscopy images showing uptake of apoVs (red) by bone marrow‐derived macrophages (BMDMs) (green) in vitro, counterstained by Hoechst (blue), and the corresponding quantification of fold changes of relative fluorescence intensity. apoV+CRT‐nAb, apoVs pre‐treated with CRT neutralizing antibody at 37℃ for 1 h. Scale bars, 25 μm. *N* = 5–6 per group. (b) Flow cytometric analysis showing uptake of apoVs by BMDMs in vitro, and the corresponding quantification of fold changes of mean fluorescence intensity (MFI). *N* = 6 per group. (C‐E) Quantitative real time polymerase chain reaction (qRT‐PCR) analysis of mRNA expression levels of tumor necrosis factor (*Tnf*) (c), interleukin 1 beta (*Il1b*) (d) and nitric oxide synthase 2, inducible (*Nos2*) (e) in cultured BMDMs, normalized to β‐actin (*Actb*), and quantification of fold changes over the Ctrl group. Ctrl, BMDMs derived from control mice; DIO, BMDMs derived from mice with diet‐induced obesity; apoV, BMDMs derived from DIO mice and treated with apoVs; apoV+CRT‐nAb, BMDMs derived from DIO mice and treated with apoVs which were pre‐treated with CRT‐nAb. *N* = 3–4 per group. (f) Enzyme‐linked immunosorbent assay (ELISA) analysis of TNF‐α in media from cultured BMDMs. *N* = 4 per group. (g‐i) qRT‐PCR analysis of mRNA expression levels of interleukin 10 (*Il10*) (g), mannose receptor, C type 1 (*Mrc1*) (h) and resistin like alpha (*Retnla*) (i) in cultured BMDMs, normalized to *Actb*, and quantification of fold changes over the Ctrl group. *N* = 3–4 per group. (j) ELISA analysis of IL‐10 in media from cultured BMDMs. *N* = 4 per group. (k) qRT‐PCR analysis of mRNA expression levels of chemokine (C‐C motif) ligand 2 (*Ccl*2) in cultured BMDMs, normalized to *Actb*, and quantification of fold changes over the Ctrl group. *N* = 3–4 per group. (l) ELISA analysis of CCL2 in media from cultured BMDMs. *N* = 4 per group. (m) Representative confocal microscopy images showing uptake of apoVs (red) by macrophages (green) in the liver, counterstained by Hoechst (blue). Scale bars, 25 μm. Data are presented as mean ± standard deviation (SD). Statistical analyses are performed by Student's *t* test with Welch correction (two‐tailed) for two group comparisons and One‐way ANOVA with Tukey's post hoc test for multiple group comparisons. *, *P* < 0.05; **, *P* < 0.01; ***, *P* < 0.001

To unravel whether surface CRT contributes to apoV efferocytosis, we blocked surface CRT with a neutralizing antibody, as stated above, and incubated them with BMDMs. As expected, the uptake of apoVs by BMDMs was significantly inhibited by surface CRT blockade, as shown by confocal microscopy and flow cytometric analyses (Figure 6(a, b)), supporting the role of surface CRT in mediating apoV efferocytosis. We then cultured DIO mice‐derived BMDMs in vitro to decipher whether surface CRT blockade would affect the functional effects of apoVs. In line with the aforementioned in vivo effects, BMDMs derived from DIO mice displayed pro‐inflammatory state (Figure 6(c‐j)), as shown by increased expression of M1 markers, *Tnf*, *Il1b* and nitric oxide synthase 2, inducible (*Nos2*), as well as decreased expression of M2 markers, *Il10*, mannose receptor, C type 1 (*Mrc1*) and resistin like alpha (*Retnla*). Similar results were observed regarding the levels of pro‐ and anti‐inflammatory factors in the supernatants. As to the production of chemokines, the mRNA level of *Ccl2* and media CCL2 concentration were significantly increased in DIO BMDMs (Figure 6(k, l)). Notably, efferocytosis of apoVs inhibited pro‐inflammation activation and chemokine production as well as promoted anti‐inflammation conversion, which was markedly suppressed by surface CRT blockade (Figure 6(c‐l)). We also systemically injected apoVs and evaluated the efficiency for efferocytosis in vivo. As shown in Figure 6(m), apoVs with surface CRT blockade were not efficiently engulfed by KCs and MoMFs, which was consistent with the in vitro assay.

**FIGURE 7:**
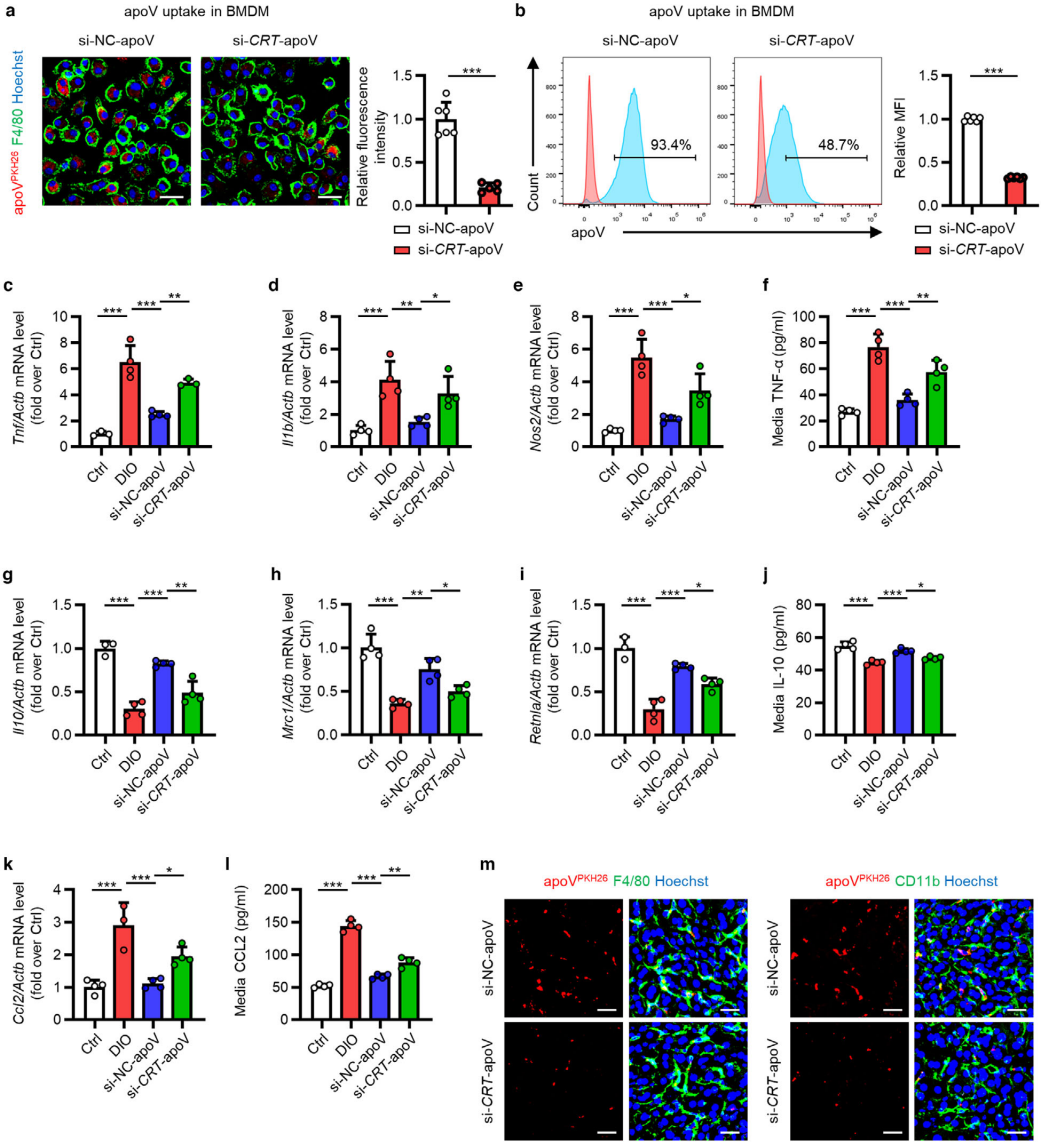
CRT downregulation inhibits functional efferocytosis of MSC‐derived apoVs by T2D macrophages in vitro. (a) Representative confocal microscopy images showing uptake of apoVs (red) by bone marrow‐derived macrophages (BMDMs) (green) in vitro, counterstained by Hoechst (blue), and the corresponding quantification of fold changes of relative fluorescence intensity. si‐NC‐apoV, apoVs derived from MSCs treated by siRNA‐negative control; si‐*CRT*‐apoV, apoVs derived from MSCs treated by siRNA‐*CRT*. Scale bars, 25 μm. *N* = 5–6 per group. (b) Flow cytometric analysis showing uptake of apoVs by BMDMs in vitro, and the corresponding quantification of fold changes of mean fluorescence intensity (MFI). *N* = 6 per group. (C‐E) Quantitative real time polymerase chain reaction (qRT‐PCR) analysis of the mRNA expression levels of tumor necrosis factor (*Tnf*) (c), interleukin 1 beta (*Il1b*) (d) and nitric oxide synthase 2, inducible (*Nos2*) (e) in cultured BMDMs, normalized to β‐actin (*Actb*), and quantification of fold changes over the Ctrl group. Ctrl, BMDMs derived from control mice; DIO, BMDMs derived from mice with diet‐induced obesity; si‐NC‐apoV, BMDMs derived from DIO mice and treated with si‐NC‐apoVs; si‐*CRT*‐apoV, BMDMs derived from DIO mice and treated with si‐*CRT*‐apoVs. *N* = 3–4 per group. (f) Enzyme‐linked immunosorbent assay (ELISA) analysis of TNF‐α in media from cultured BMDMs. *N* = 4 per group. (G‐I) qRT‐PCR analysis of mRNA expression levels of interleukin 10 (*Il10*) (g), mannose receptor, C type 1 (*Mrc1*) (h) and resistin like alpha (*Retnla*) (i) in cultured BMDMs, normalized to *Actb*, and quantification of fold changes over the Ctrl group. *N* = 3–4 per group. (j) ELISA analysis of IL‐10 in media from cultured BMDMs. *N* = 4 per group. (k) qRT‐PCR analysis of mRNA expression levels of chemokine (C‐C motif) ligand 2 (*Ccl*2) in cultured BMDMs, normalized to *Actb*, and quantification of fold changes over the Ctrl group. *N* = 3–4 per group. (l) ELISA analysis of CCL2 in media from cultured BMDMs. *N* = 4 per group. (m) Representative confocal microscopy images showing uptake of apoVs (red) by macrophages (green) in the liver, counterstained by Hoechst (blue). Scale bars, 25 μm. Data are presented as mean ± standard deviation (SD). Statistical analyses are performed by Student's *t* test (two‐tailed) or Student's *t* test with Welch correction (two‐tailed) for two group comparisons and One‐way ANOVA with Tukey's post hoc test for multiple group comparisons. *, *P* < 0.05; **, *P* < 0.01; ***, *P* < 0.001

To further verity the role of CRT, we downregulated the expression of CRT through siRNA technology and incubated them with BMDMs. Compared to si‐NC‐apoVs, the uptake of si‐*CRT*‐apoVs by BMDMs was markedly suppressed (Figure 7(a, b)). As to the functional effects, efferocytosis of si‐NC‐apoVs downregulated the expression of M1 markers and pro‐inflammatory cytokine level while increased the expression of M2 markers and anti‐inflammatory cytokine level, which was significantly inhibited by knockdown of *CRT* (Figure 7(c‐j)). Furthermore, the suppressive effects on the production of CCL2 by si‐NC‐apoVs engulfment was reversed by *CRT* knockdown, as shown by detection of mRNA level and media concentration (Figure 7(k, l)). Furthermore, in vivo assay also showed that *CRT* knockdown inhibited the engulfment of apoVs by KCs and MoMFs (Figure 7(m)), supporting contribution of CRT to apoV efferocytosis in vivo. These data suggest that CRT mediates efferocytosis of apoVs to modulate T2D macrophage in vitro.

### 3.6 CRT mediates efferocytosis of MSC‐derived apoVs for T2D macrophage modulation in vivo

**FIGURE 8:**
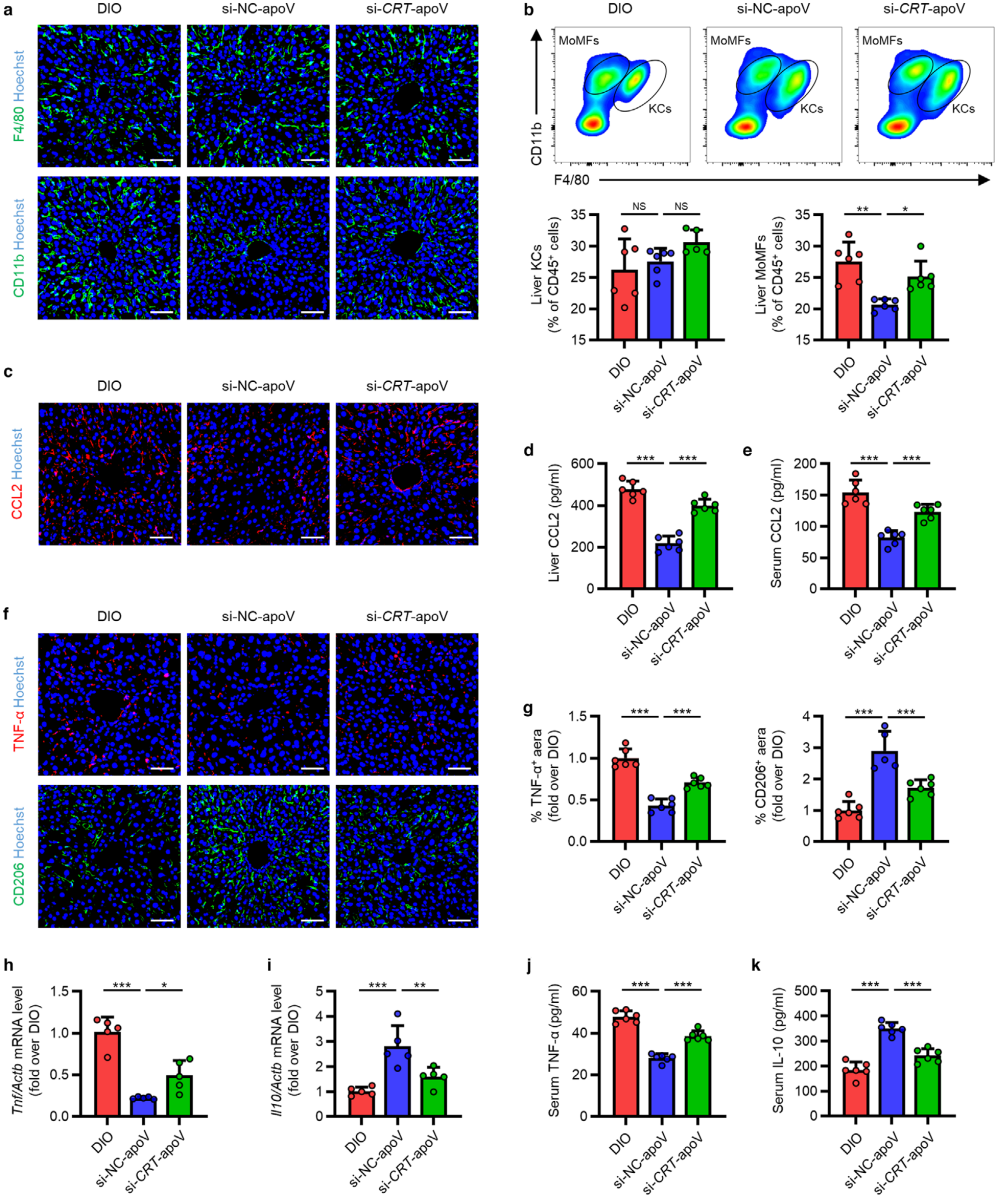
CRT mediates efferocytosis of MSC‐derived apoVs to modulate T2D liver macrophages in vivo. (a) Representative immunofluorescent (IF) staining images of F4/80 (green) and CD11b (green) in the liver, counterstained by Hoechst (blue). DIO, mice with diet‐induced obesity; si‐NC‐apoV, DIO mice treated by apoVs derived from MSCs transfected by siRNA‐negative control; si‐*CRT*‐apoV, DIO mice treated by apoVs derived from MSCs transfected by siRNA‐*CRT*. Scale bars, 50 μm. (b) Flow cytometric analysis and the corresponding quantification of the percentages of KCs and MoMFs in hepatic CD45^+^ cells. KCs, Kupffer cells; MoMFs, monocyte‐derived macrophages. *N* = 5–6 per group. (c) Representative IF staining images of chemokine (C‐C motif) ligand 2 (CCL2) (red) in the liver, counterstained by Hoechst (blue). Scale bars, 50 μm. (d and e) ELISA analysis of CCL2 in liver lysate (d) and serum (e). *N* = 6 per group. (f and g) Representative IF staining images of tumor necrosis factor‐alpha (TNF‐α) (red) and CD206 (green) in the liver, counterstained by Hoechst (blue), and the corresponding quantification of fold changes over the DIO group. Scale bars, 50 μm. *N* = 5–6 per group. (h and i) Quantitative real time polymerase chain reaction (qRT‐PCR) analysis of mRNA expression levels of *Tnf* (h) and interleukin 10 (*Il10*) (i) in the liver, normalized to β‐actin (*Actb*), and quantification of fold changes over the DIO group. *N* = 5 per group. (j and k) Enzyme‐linked immunosorbent assay (ELISA) analysis of TNF‐α (j) and IL‐10 (k) in serum. *N* = 6 per group. Data are presented as mean ± standard deviation (SD). Statistical analyses are performed by One‐way ANOVA with Tukey's post hoc test or Kruskal‐Wallis H test. *, *P* < 0.05; **, *P* < 0.01; ***, *P* < 0.001; NS, *P* > 0.05

Based on the observation of cultured macrophages, we proceeded to investigate the role of CRT in vivo. We injected si‐NC‐apoVs and si‐*CRT*‐apoVs into DIO mice and detected the changes of liver macrophages. While DIO mice with efferocytosis of si‐NC‐apoVs displayed significant inhibition of MoMF infiltration in the liver, such effects were suppressed in DIO mice receiving si‐*CRT*‐apoVs, as demonstrated by IF staining and flow cytometric analyses of macrophage markers (Figure 8(a, b)). Consistently, the level of CCL2 in the liver and serum, the percentage of peripheral blood monocytes as well as the percentage of injected monocytes migrating to the liver were higher in DIO mice with inadequate efferocytosis of apoVs due to deficiency of CRT (si‐*CRT*‐apoV group) (Figure 8(c‐e), Supplementary Figure 3(C, D)). As to the changes of liver macrophage polarization, internalization of si‐NC‐apoVs markedly inhibited M1 marker expression and promoted M2 marker expression in DIO mice, while insufficient efferocytosis of si‐*CRT*‐apoVs induced by knockdown of *CRT* showed reduced effects (Figure 8(f‐i)). Similar results were observed regarding serum pro‐ and anti‐inflammatory factors (Figure 8(j, k)). Together, these data indicate that CRT mediates efferocytosis of MSC‐derived apoVs to alleviate macrophage infiltration and activation in T2D liver.

### 3.7 CRT‐mediated efferocytosis of MSC‐derived apoVs contributes to T2D therapy

**FIGURE 9:**
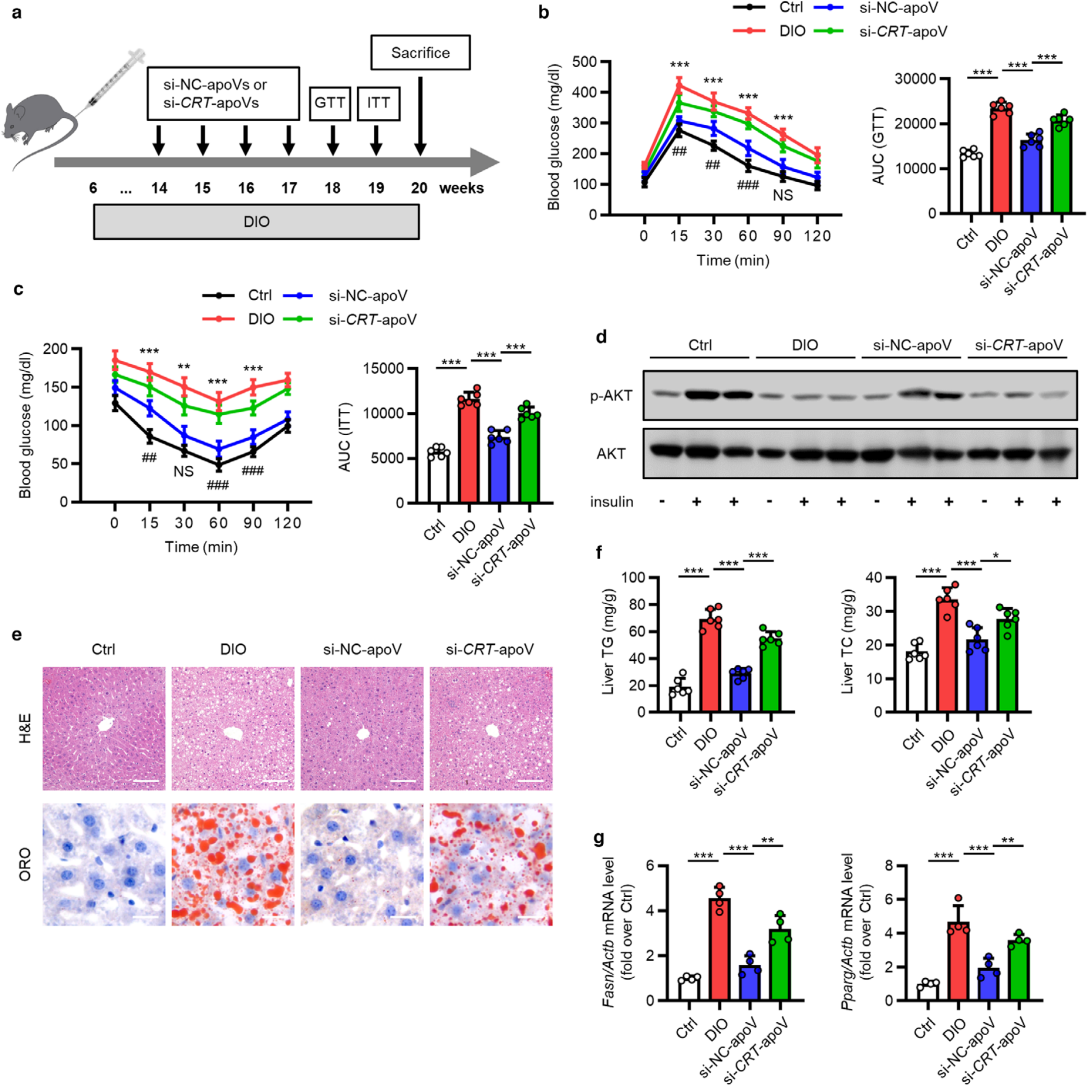
CRT‐mediated efferocytosis of MSC‐derived apoVs contributes to T2D therapy. (a) Schematic diagram indicating the study design of apoV efferocytosis‐mediated treatment for T2D in C57 mice. DIO, diet‐induced obesity; si‐NC‐apoVs, apoVs derived from MSCs treated by siRNA‐negative control; si‐*CRT*‐apoVs, apoVs derived from MSCs treated by siRNA‐*CRT*; GTT, glucose tolerance test; ITT, insulin tolerance test. (b) Blood glucose levels during GTT and quantification of area under the curve (AUC). Ctrl, control mice; DIO, mice with DIO; si‐NC‐apoV, DIO mice treated by si‐NC‐apoVs; si‐*CRT*‐apoV, DIO mice treated by si‐*CRT*‐apoVs. *, comparison between DIO and si‐NC‐apoV; ^#^, comparison between si‐NC‐apoV and si‐*CRT*‐apoV. *N* = 6 per group. (c) Blood glucose levels during ITT and quantification of AUC. *, comparison between DIO and si‐NC‐apoV; ^#^, comparison between si‐NC‐apoV and si‐*CRT*‐apoV. *N* = 6 per group. (d) Western blotting analysis of the expression levels of phosphorylated AKT (p‐AKT) and AKT in liver tissues. (e) Representative haematoxylin and eosin (H&E) and oil red O (ORO) staining images of liver tissues. Scale bars, 100 μm (top) and 25 μm (bottom). (f) Quantification of liver triglyceride (TG) and total cholesterol (TC) levels. *N* = 6 per group. (g) Quantitative real time polymerase chain reaction (qRT‐PCR) analysis of mRNA expression levels of fatty acid synthase (*Fasn*) and peroxisome proliferator‐activated receptor gamma (*Pparg*) in the liver, normalized to β‐actin (*Actb*), and quantification of fold changes over the Ctrl group. *N* = 4 per group. Data are presented as mean ± standard deviation (SD). Statistical analyses are performed by One‐way ANOVA with Tukey's post hoc test or Kruskal‐Wallis H test. *, *P* < 0.05; **, *P* < 0.01; ***, *P* < 0.001; ^##^, *P* < 0.01; ^###^, *P* < 0.001; NS, *P* > 0.05

T2D is a global healthcare challenge with huge social and economic burden, and insulin resistance is a central etiological factor (Roden & Shulman, 2019; Saeedi et al., 2019). As mentioned before, infiltration and activation of macrophages is a key contributor to hepatic insulin resistance and hepatic steatosis (Kazankov et al., 2019; Lee et al., 2018; Morinaga et al., 2015). We intended to explore whether efferocytosis of MSC‐derived apoVs could alleviate T2D. In the DIO‐induced T2D model, we established a sequential injection protocol of apoVs with an interval of 1 week (Figure 9(a)). As expected, compared to control mice, HFD feeding led to impaired glucose tolerance and insulin sensitivity in DIO mice, as measured by GTT and ITT (Figure 9(b, c)). Insulin‐stimulated phosphorylation of AKT, an index for insulin signalling, was also intensively reduced in DIO liver (Figure 9(d)). DIO mice displayed severe hepatic steatosis, as detection by H&E and ORO staining as well as measurement of TG and TC levels in the liver (Figure 9(e, f)). The mRNA levels of adipogenic genes, fatty acid synthase (*Fasn*) and peroxisome proliferator‐activated receptor gamma (*Pparg*), in the liver were upregulated (Figure 9(g)). Importantly, efferocytosis of si‐NC‐apoVs led to significant improvement of glucose tolerance and insulin sensitivity in DIO mice, whereas such effects were inhibited in si‐*CRT*‐apoV group with insufficient efferocytosis of apoVs (Figure 9(b, c)). Consistently, insulin‐stimulated phosphorylation of AKT was recovered by uptake of si‐NC‐apoVs but not si‐*CRT*‐apoVs (Figure 9(d)). Histological evaluation *via* H&E and ORO staining showed that si‐NC‐apoV efferocytosis alleviated hepatic steatosis, which was diminished by insufficient efferocytosis due to knockdown of *CRT* (Figure 9(e)). Moreover, TG and TC concentration as well as adipogenic gene levels in the liver demonstrated similar results (Figure 9(f, g)). Collectively, these results reveal that efferocytosis of MSC‐derived apoVs is able to improve glucose tolerance, ameliorate insulin resistance and alleviate hepatic steatosis in T2D mice which depends on the presence of CRT.

Considering that apoV efferocytosis restored liver macrophage homeostasis of T2D mice and alleviated metabolic abnormalities, we took a further step to decipher whether physiological apoVs contributed to maintaining macrophage homeostasis and whether apoV deficiency promoted T2D progression. For this purpose, we took advantage of apoptosis‐deficient *Fas^mut^* mice (previously referred to as *Fas^lpr^* mice) which are characterized by functional deficiency of Fas, a well‐characterized cell‐surface receptor that mediates apoptosis upon binding to Fas ligand (FasL) (Strasser et al., 2009; Wajant, 2002). We have previously reported that the production of apoVs is markedly reduced in *Fas^mut^* mice (Liu et al., 2018). Here, we replenished MSC‐derived apoVs into *Fas^mut^* mice *via* the tail vein. Compared with WT mice, the number of both liver KCs and MoMFs were markedly increased in *Fas^mut^* mice (Supplementary Figure 4(A)), indicating that lack of apoVs resulted in both proliferation of resident KCs and infiltration of MoMFs in the liver. Notably, the increase of these two populations was significantly suppressed by systemic administration of apoVs (Supplementary Figure 4(A)). Such results primed us to further decipher the effects of apoV deficiency and replenishment under pathological condition. We established HFD‐induced T2D model in *Fas^mut^* mice and investigated the protective effects *via* intravenously injecting apoVs biweekly since the beginning of the modelling (Supplementary Figure 4(B)). In comparison to WT mice, HFD feeding led to more severe impairment of glucose tolerance and insulin sensitivity in *Fas^mut^* mice (Supplementary Figure 4(C, D)), suggesting an aggravating role of apoV deficiency in T2D progression. Exogenous apoV treatment significantly protected *Fas^mut^* mice from HFD‐induced glucose intolerance and insulin resistance (Supplementary Figure 4(C, D)). Thus, physiological apoV deficiency exacerbates T2D progression which can be mitigated by apoV replenishment.

**FIGURE 10:**
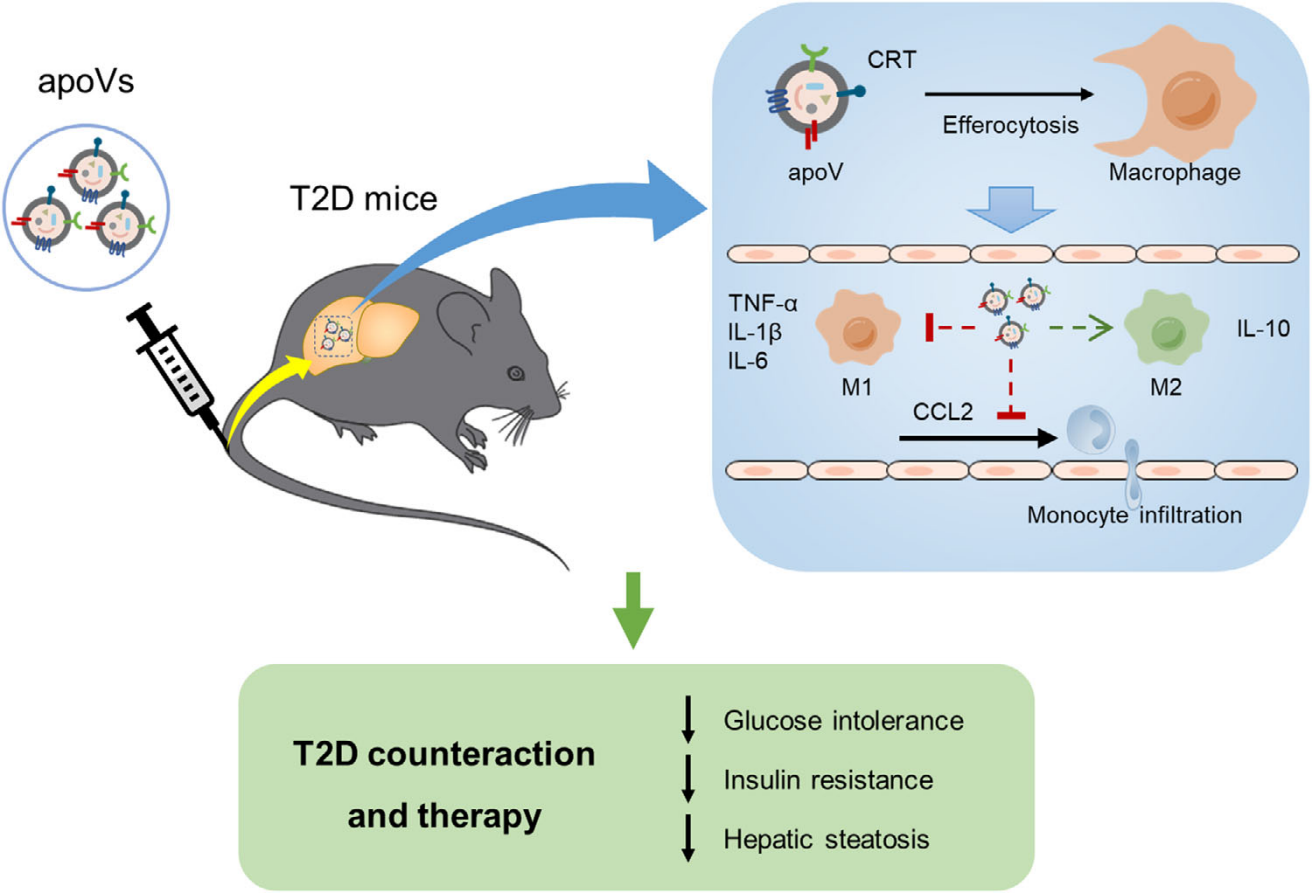
Schematic diagram showing the synopsis of the findings. Systemic infusion of MSC‐derived apoVs modulates liver macrophage function *via* calreticulin (CRT)‐mediated efferocytosis, thus providing a promising therapy for T2D. M1, pro‐inflammatory macrophages; M2, anti‐inflammatory macrophages; TNF‐α, tumor necrosis factor‐alpha; IL‐1β, interleukin‐1 beta; IL‐6, interleukin‐6; IL‐10, interleukin‐10; CCL2, chemokine (C‐C motif) ligand 2

Taken together, we summarize the main in vivo findings as follows: CRT serves as a critical ‘eat‐me’ signal to mediate apoV efferocytosis by liver macrophages after systemic infusion; apoV engulfment alleviates macrophage infiltration and promotes macrophage polarization towards anti‐inflammation phenotype; functional efferocytosis of apoVs provides an effective strategy for T2D counteraction and therapy (Figure 10).

## 4 DISCUSSION

While EVs from viable cells have attracted substantial attention within recent years, our understanding of apoVs produced by apoptotic cells is still very limited. The formation of apoVs, in particular the packaging of cargos, depends on the type of parental cells, thus apoVs generated by different cell types may possess distinct functions (Canbay, 2003; Ma et al., 2020; Pavlyukov et al., 2018). MSCs are attractive candidate cells for the treatment of multiple diseases (Bianco et al., 2013; Galipeau & Sensébé, 2018; Yin et al., 2019). In this study, we have collected MSC‐derived apoVs and characterized them *via* multiple approaches. The features of MSC‐derived apoVs have been shown to be heterogeneous in size with diameters less than 1 μm, whereas universally possess PtdSer, suggesting the predominant origin of plasma membrane exposed during apoptosis (Segawa & Nagata, 2015). These apoVs also encapsulate apoptosis‐featured proteins, including BAX and Caspase‐3, the apoptotic inducer and executioner, respectively (Nagata, 2018; Walensky, 2019). Proteins capable of regulating apoptosis are also found in apoVs, such as RTN4, a molecule participating in all three apoptosis signalling pathways (Chen et al., 2010), and GSN, a substrate for caspase‐3 and a physiological effector of morphologic change during apoptosis (Kothakota, 1997; Springer et al., 1999). These findings have promoted our understanding of MSC‐derived apoVs and provided reference for future studies.

Macrophage polarization is crucial for tissue repairing and homeostasis maintenance (Murray, 2017). Particularly, M2 macrophages have been described to regulate the resolution phase of inflammation and the repair of damaged tissues (Smith et al., 2017). In this study, among the enriched proteins in apoVs, some possess the potential to induce polarization of macrophages towards the M2 phenotype, which provides a molecular basis for the biological effects of apoVs. VASP, the actin‐associated protein involved in a range of processes, has been revealed as a key regulator of macrophage M2 polarization after ischemia and in HFD‐induced hepatic inflammation (Lee et al., 2015). PRKAR2A, the regulatory subunit of the cAMP‐dependent protein kinases, suppresses JAK2 signalling to mediate macrophage M2 polarization for inflammatory resolution (Kong et al., 2016). CRYAB, a small heat‐shock protein, has been demonstrated as a potent negative regulator of inflammatory pathways involved in macrophage activation (Chen et al., 2010; Fiedler et al., 2019). Based on these and other proteins, MSC‐derived apoVs exert extensive regulatory effects on macrophages at the transcription level, which contributes to macrophage polarization towards the anti‐inflammation phenotype in T2D treatment. Additional experiments are required so as to decipher the specific functions of enriched proteins.

As to the in vivo fate of apoVs, we have found that systemically infused apoVs are mainly engulfed by liver macrophages, which are the professional phagocytes for the clearance of apoptotic cells (Boada‐Romero et al., 2020; Doran et al., 2020; Morioka et al., 2019). Notably, we have confirmed that an important ‘eat‐me’ signal, CRT, is exposed on the surface of apoVs and can be used as a tool for fractionating apoV subpopulation through an antibody‐based affinity purification method. Through using neutralizing antibody and siRNA approaches to suppress CRT, we have verified that CRT indeed mediates apoV uptake by macrophages both in vitro and in vivo. We have also revealed that CRT‐based engulfment of MSC‐derived apoVs leads to alleviation of infiltration and activation of diseased liver macrophages in vivo. Taken together, these findings add to the current knowledge on the mechanisms and biological effects of apoV efferocytosis. Intriguingly, surface exposure of CRT on cancer cells has been recognized as a characteristic of immunogenic cell death (Obeid et al., 2007). Therefore, it is likely that CRT‐mediated efferocytosis of different cells or cellular derivatives may trigger distinct responses of phagocytes. Moreover, considering that CRT is a highly negatively charged protein binding to a variety of proteins/molecules (Eggleton et al., 2016), the ligands/receptors interacting with CRT may affect the downstream effects. More studies are needed so as to further clarify the signalling molecules and pathways involved in functional efferocytosis of apoVs.

T2D is a major threat to global public health, with increasing prevalence as well as high morbidity and mortality (Castegna et al., 2020; Saeedi et al., 2019; Zheng et al., 2018). Due to the pathogenic role of macrophage‐mediated chronic inflammation in T2D, targeting and reprogramming of macrophages has provided an effective therapeutic strategy (Castegna et al., 2020; Mcnelis & Olefsky, 2014). In this study, we have provided a proof‐of‐concept study that MSC‐derived apoVs possess macrophage‐targeting feature and immunomodulatory ability. Our results are in line with previous studies showing that thymocyte‐derived apoVs promote macrophage production of TGF‐β to ameliorate experimental colitis (Chen et al., 2019) and platelet apoVs induce monocytic cells to differentiate into M2 macrophages (Vasina et al., 2011). We have verified that the chronic inflammation condition in T2D was improved by apoV infusion, through which apoV administration efficaciously alleviated glucose intolerance, insulin resistance and hepatic steatosis in T2D. Considering that apoVs are easily obtained and stored, with low immunological rejection and neoplastic transformation risk, our findings will provide a potent rationale for translational paradigms and promote establishment of optimized cell‐free therapies.

In conclusion, our study extends the current understanding of apoVs and sheds light on the key role played by efferocytosis of apoVs in macrophage modulation, thus paving the way to new paradigms in T2D therapy.

## CONFLICTS OF INTEREST

The authors declare no conflicts of interest.

## AUTHOR CONTRIBUTIONS

Chenxi Zheng, Bingdong Sui and Xiao Zhang contributed equally to the experimental performing, data acquisition and analysis, and manuscript drafting. Jiachen Hu, Ji Chen and Jin Liu contributed to animal experiments. Di Wu, Qingyuan Ye and Lei Xiang contributed to data analysis and interpretation. Xinyu Qiu and Siying Liu contributed to flow cytometry analysis. Zhihong Deng and Jun Zhou contributed to data interpretation. Shiyu Liu, Songtao Shi and Yan Jin contributed to the study conception and design, data interpretation and manuscript revision. All authors have read and approved the current version of the manuscript.

## Supporting information

Supporting information.

Supporting information.

Supporting information.

Supporting information.

Supporting information.

Supporting information.

Supporting information.

Supporting information.

Supporting information.

Supporting information.

Supporting information.
