# Supplementary material for: Apoptotic vesicles restore liver macrophage homeostasis to counteract type 2 diabetes
Source: J Extracell Vesicles. 2021 May 24;10(7):e12109. doi: 10.1002/jev2.12109 (PMC8144839; doi:10.1002/jev2.12109)
Supplement: Supplementary file 1 — Supporting information. [file JEV2-10-e12109-s011.docx]

**Supplementary figure legends**

**Figure S1. Characterization of mesenchymal stem cells (MSCs).**

(A) Flow cytometric analysis of the surface markers of MSCs.

(B) Representative images showing the colony formation ability, the spindle shape, and the osteogenic differentiation as well as adipogenic differentiation of MSCs. Undifferentiated MSCs with alizarin red S and oil red O (ORO) staining were also depicted. Scale bars, 500 μm (left) and 100 μm (medium and right).

(C) Representative bright field and terminal deoxynucleotidyl transferase dUTP nick end labeling (TUNEL) (red) staining images of MSCs and staurosporine (STS)-induced apoptotic MSCs, counterstained by Hoechst (blue). Scale bars, 200 μm (bright field) and 50 μm (TUNEL staining).

**Figure S2. Isolation, identification and uptake of MSC-derived apoptotic vesicles (apoVs).**

(A) Schematic diagram indicating the protocol for isolation of MSC-derived apoVs. STS, staurosporine.

(B) Representative wide field transmission electron microscope (TEM) image showing the morphology of apoVs. Scale bar, 500 nm.

(C) Representative cryo-electron microscopy (Cryo-EM) images showing the morphology of apoVs. Scale bars, 500 nm (low magnification) and 125 nm (high magnification).

(D) Representative images showing the captured frame of apoVs and filtered PBS during nanoparticle tracking analysis (NTA).

(E) Flow cytometric analysis of the expression levels of F4/80 and CD11b in bone marrow-derived macrophages (BMDMs) and representative confocal microscopy images showing uptake of PKH26-labeled apoVs (red) by BMDMs (green), counterstained by Hoechst (blue) and focusing on various positions of Z-axis. Scale bars, 20 μm.

(F) *In vivo* imaging analysis and confocal microscopy images showing *in vivo* distribution of apoVs, as well as quantitation of radiant efficiency and number of PKH26^+^ cells. HPF, high power field. Scale bars, 50 μm. *N* = 3-6 per group.

Data are presented as mean ± standard deviation (SD). Statistical analyses are performed by One-way ANOVA with Tukey’s post hoc test. ***, *P* < 0.001.

**Figure S3. Calreticulin (CRT) mediates efferocytosis of MSC-derived apoptotic vesicles (apoVs) to modulate type 2 diabetes (T2D) liver macrophage accumulation.**

(A) Mesenchymal stem cells (MSCs) were transfected with three siRNA sequences targeting *CRT* for 48 h, and the expression of *CRT* gene was detected by quantitative real time polymerase chain reaction (qRT-PCR), normalized to β-actin (*ACTB*) and quantified as fold changes over the si-NC group. si-NC, siRNA-negative control; si-*CRT*, siRNA-*CRT*. *N* = 3 per group.

(B) Western blotting analysis of CRT protein levels in apoVs, indicating knockdown of *CRT*. si-NC-apoV, apoVs derived from MSCs treated by si-NC; si-*CRT*-apoV, apoVs derived from MSCs treated by the third sequence of si-*CRT* in (A).

(C) Flow cytometric analysis and the corresponding quantification of the percentages of monocytes in the peripheral blood CD45^+^ cells. *N* = 6 per group.

(D) Flow cytometric analysis and the corresponding quantification of the percentages of PKH67-labeled monocytes migrating to the liver. *N* = 4 per group.

Data are presented as mean ± standard deviation (SD). Statistical analyses are performed by One-way ANOVA with Tukey’s post hoc test. *, *P* < 0.05; **, *P* < 0.01; ***, *P* < 0.001.

**Figure S4. Endogenous apoV deficiency aggravates T2D which is recovered by the infusion of MSC-derived apoVs.**

(A) Representative immunofluorescent (IF) staining images of F4/80 (green) and CD11b (green) in the liver, counterstained by Hoechst (blue), and the corresponding quantification of fold changes over WT group. WT, wild type; *Fas^mut^*, Fas mutation-induced apoptosis-deficient mice with physiological apoV deficiency; *Fas^mut^*+apoV, *Fas^mut^* mice with apoV replenishment. Scale bars, 50 μm. *N* = 3 per group.

(B) Schematic diagram indicating the study design of apoV replenishment and T2D modeling in *Fas^mut^* mice. DIO, diet-induced obesity; GTT, glucose tolerance test; ITT, insulin tolerance test.

(C) Blood glucose levels during GTT and quantification of area under the curve (AUC). WT-DIO, WT mice with DIO; *Fas^mut^*-DIO, *Fas^mut^* mice with DIO; *Fas^mut^*-DIO+apoV, *Fas^mut^* mice with DIO and apoV replenishment. *, comparison between WT-DIO and *Fas^mut^*-DIO; ^#^, comparison between *Fas^mut^*-DIO and *Fas^mut^*-DIO+apoV. *N* = 4 per group.

(D) Blood glucose levels during ITT and quantification of AUC. *, comparison between WT-DIO and *Fas^mut^*-DIO; ^#^, comparison between *Fas^mut^*-DIO and *Fas^mut^*-DIO+apoV. *N* = 4 per group.

Data are presented as mean ± standard deviation (SD). Statistical analyses are performed by One-way ANOVA with Tukey’s post hoc test or Welch's ANOVA with Tamhane's T2 post hoc test. *, *P* < 0.05; **, *P* < 0.01; ***, *P* < 0.001; ^#^, *P* < 0.05; ^##^, *P* < 0.01; ^###^, *P* < 0.001; NS, *P* > 0.05.

**Supplementary table legends**

**Table S1. List of all the proteins identified during proteomic analysis.**

**Table S2. Quantity of RNA sequencing (RNA-seq) data output.**

**Table S3. List of all the genes identified during RNA-seq analysis.**

**Table S4. Primers for quantitative real time polymerase chain reaction (qRT-PCR).**

**Table S5. List of the key apoptotic markers** **that are significantly upregulated in apoptotic vesicles (apoVs) during proteomic analysis.**

**Table S6. List of the** **proteins with the potential to induce M2 polarization of macrophages that are significantly upregulated in apoVs during proteomic analysis.**
