# Supplementary figures and images for: Apoptotic vesicles restore liver macrophage homeostasis to counteract type 2 diabetes
Source: J Extracell Vesicles. 2021 May 24;10(7):e12109. doi: 10.1002/jev2.12109 (PMC8144839; doi:10.1002/jev2.12109)

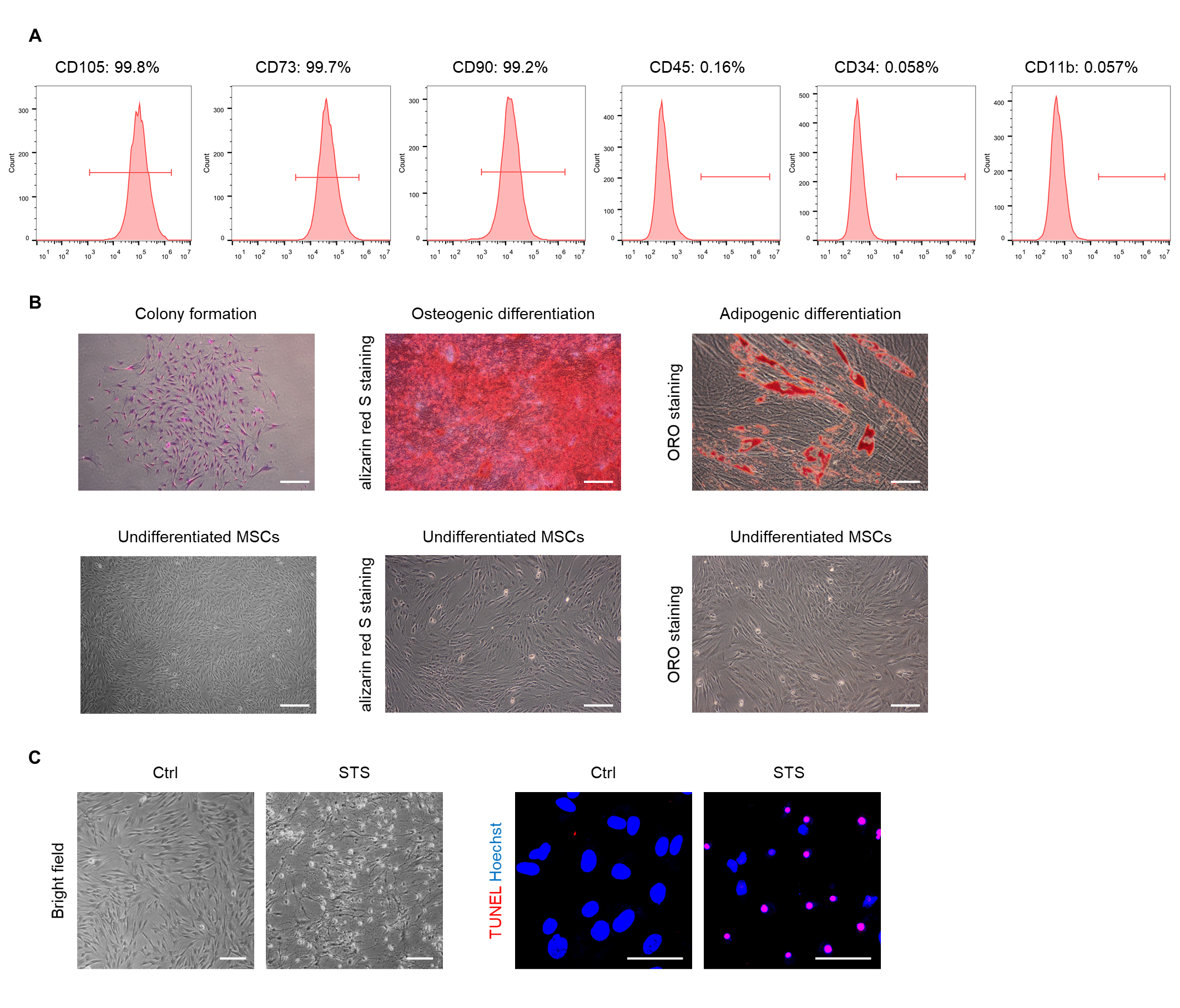

Supplement: Supplementary file 2 — Supporting information. [file JEV2-10-e12109-s008.tif]

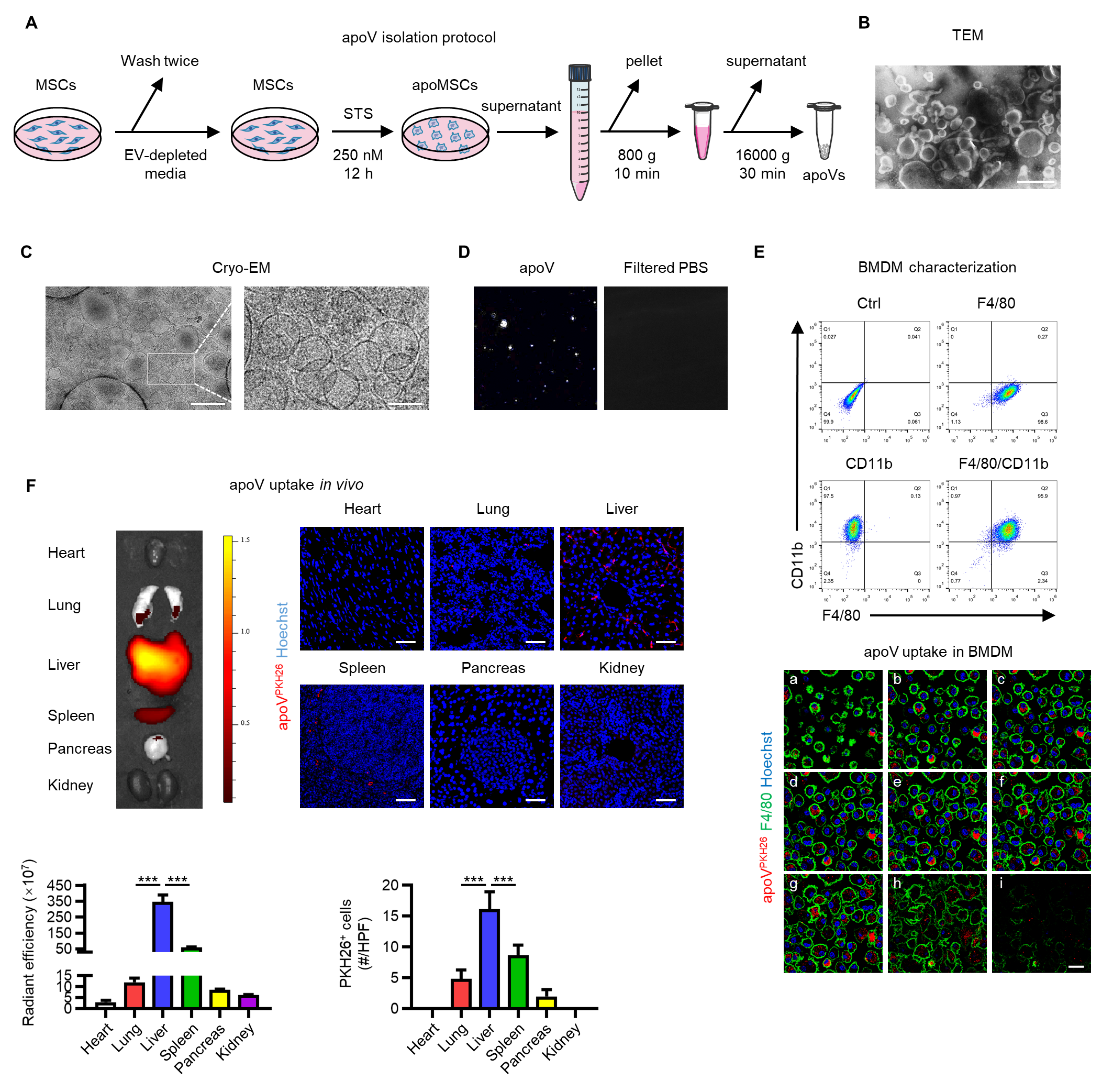

Supplement: Supplementary file 3 — Supporting information. [file JEV2-10-e12109-s004.tif]

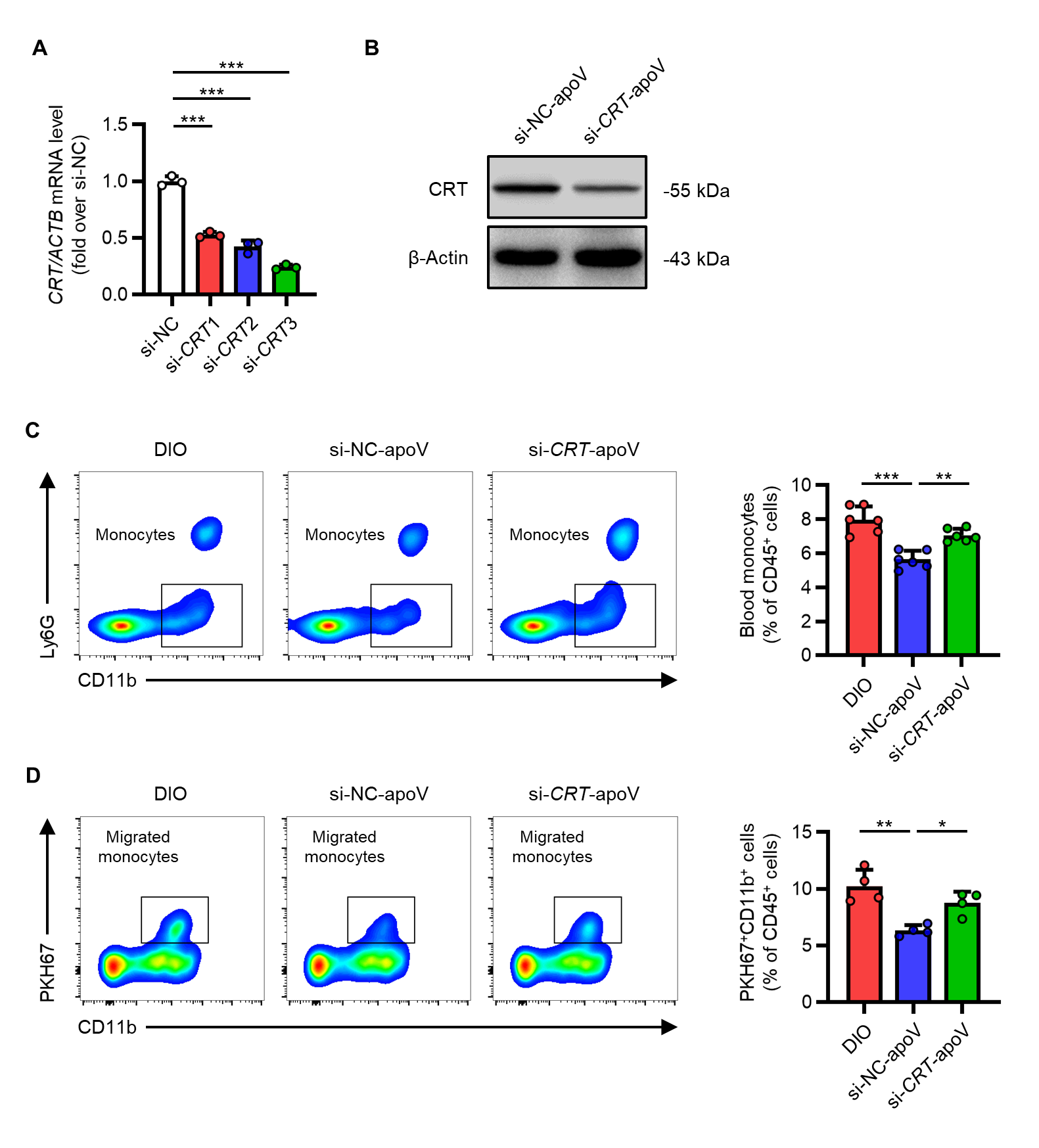

Supplement: Supplementary file 4 — Supporting information. [file JEV2-10-e12109-s003.tif]

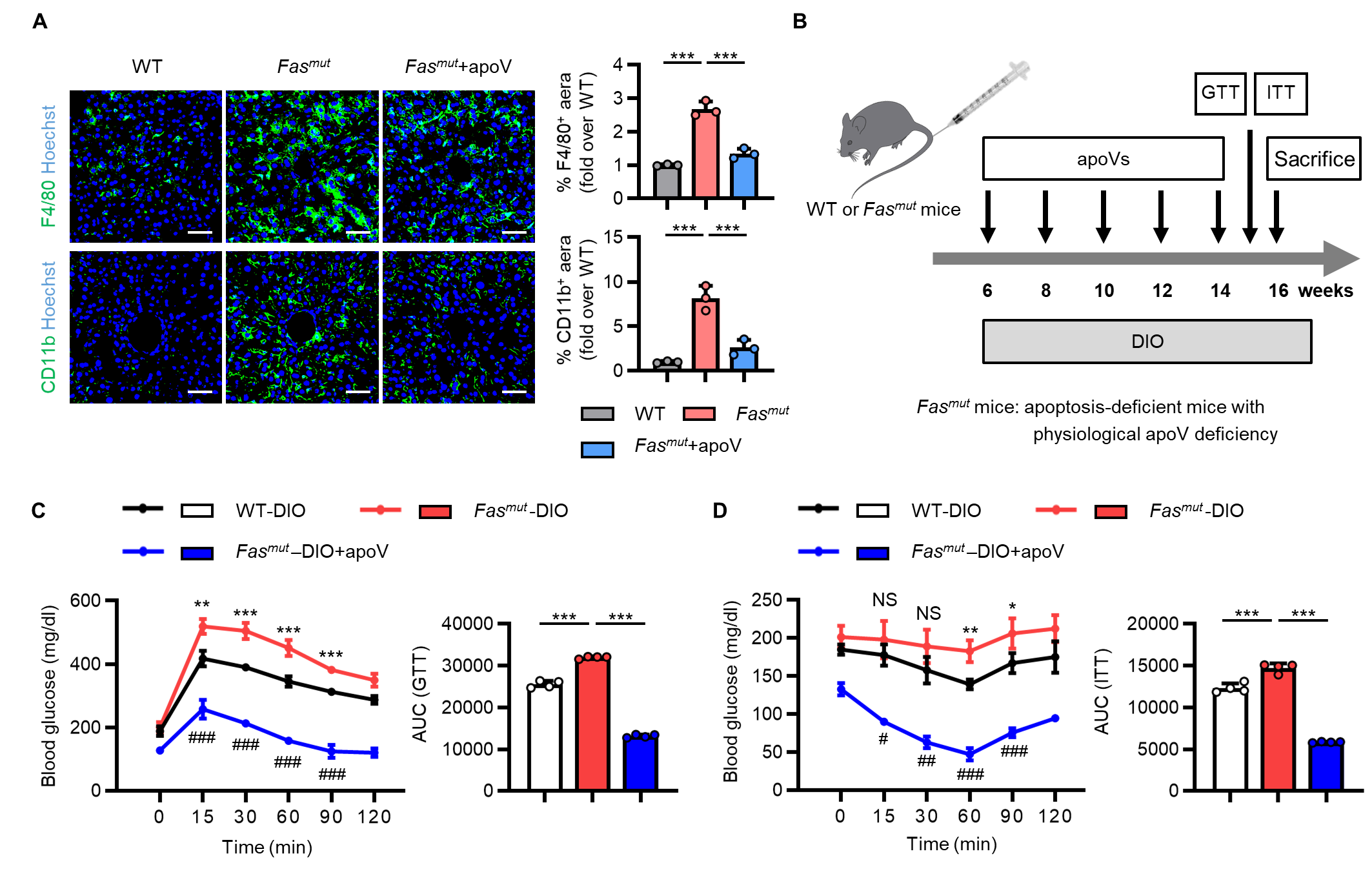

Supplement: Supplementary file 5 — Supporting information. [file JEV2-10-e12109-s001.tif]
